# Water Quality, Toxicity and Diversity of Planktonic and Benthic Cyanobacteria in Pristine Ancient Lake Khubsugul (Hövsgöl), Mongolia

**DOI:** 10.3390/toxins15030213

**Published:** 2023-03-10

**Authors:** Olga I. Belykh, Ekaterina G. Sorokovikova, Irina V. Tomberg, Galina A. Fedorova, Anton V. Kuzmin, Andrey Yu. Krasnopeev, Maria Yu. Suslova, Sergey A. Potapov, Tatiana I. Belykh, Jadambaa Norovsuren, Agnia D. Galachyants, Irina V. Tikhonova

**Affiliations:** 1 Limnological Institute of the Siberian Branch of the Russian Academy of Sciences, 3 Ulan-Batorskaya Str., Irkutsk 664033, Russia; 2 Institute for Culture, Social Communication and Information Technology, Baikal State University, 11 Lenin Str., Irkutsk 664003, Russia; 3 Institute of Biology of the Mongolian Academy of Sciences, 54B Peace Avenue, Bayanzurkh District, Ulaanbaatar 13330, Mongolia

**Keywords:** Lake Khubsugul, cyanobacteria, microcystins, coliform bacteria, high-throughput sequencing, enzyme-linked immunosorbent assay

## Abstract

For the first time, microcystin-producing cyanobacteria have been detected in Khubsugul, which is ancient, pristine and one of the world’s largest lakes. The microcystin synthetase genes belonged to the genera *Nostoc*, *Microcystis* and possibly *Snowella* spp. No microcystins were found in the water of the lake. Using the HPLC-HRMS/TOF, five microcystin congeners were identified in biofilms from stony substrates sampled in the coastal zone. The concentration of microcystins in biofilms was low: 41.95 µg g^−1^ d. wt. by ELISA and 55.8 µg g^−1^ d. wt. using HPLC. The taxonomic composition of planktonic and benthic cyanobacterial communities was determined by means of microscopy and high-throughput sequencing of 16S rDNA amplicons. Nostocales cyanobacteria dominated benthos of Lake Khubsugul and Synechococcales—plankton. The abundance of cyanobacteria was low both in plankton and benthos; there was no mass development of cyanobacteria. Hydrochemical and microbiological analyses showed that the water in the lake was clean; the number of faecal microorganisms was significantly below the acceptable guideline values. Hydrochemical and hydrophysical parameters, and the concentration of chlorophyll *a*, were low and within the range of values recorded in the 1970s to 1990s, and corresponded to the oligotrophic state of the lake. There were no signs of anthropogenic eutrophication of the lake and no conditions for the cyanobacterial blooms.

## 1. Introduction

Cyanobacteria are a successful group of morphologically diverse prokaryotes that are widespread in aquatic ecosystems with different trophic statuses, where they play an important role as primary producers, nitrogen-fixing bacteria and as a food resource. Cyanobacteria are one of the most ancient organisms on the Earth: their fossils are found in the Precambrian deposits (about 3.5 billion years old) [1,2].

Under favourable conditions, such as nutrient availability, high temperatures and luminosity, CO_2_ accessibility, high pH, a low N:P ratio, bottom-up influence, low water mixing and cyanobacteria multiply intensively, forming planktonic and benthic blooms in water bodies [3,4,5,6,7,8]. Their unique metabolism is also used for biotechnological purposes [9,10].

The high density of cyanobacteria leads to some negative phenomena in water bodies: for example, lower water transparency, growth of biomass, high oxygen consumption and the formation of anaerobic zones, unpleasant odours and a decrease in the biodiversity of aquatic organisms. Furthermore, intense cyanobacterial blooms seriously jeopardise human and animal life and health, since many cyanobacterial species synthesise toxins [3,4,5,6,7,8,11,12]. Approximately 75% of cyanobacterial blooms in water are toxic [13]. In recent decades, the frequency, occurrence and duration of cyanobacterial blooms, including toxic ones, have increased significantly. Cyanobacterial blooms were recorded not only in highly productive water bodies, but also in oligotrophic and oligo-mesotrophic lakes, including large and deep ones: for example, lakes Superior and Baikal. In Lake Superior, the near-shore and offshore zones experienced blooms of non-toxic *Dolichospermum lemmermannii* in summer [14,15]. Toxin-producing benthic and planktonic cyanobacteria develop in bays and coastal zones of Lake Baikal [16,17,18,19,20].

Microcystins (MCs), cyclic heptapeptides that inhibit protein phosphatase type 1 and 2A in liver cells and eventually cause liver failure, are the best known and most widespread cyanobacterial toxins in fresh waters. Many reviews discuss their general structure, structural MC congeners, intracellular and extracellular functions, as well as their role as MC producers and their potential (eco)toxicological risk and human health aspects [3,4,5,21,22,23,24,25,26,27].

Currently, more than 279 MC congeners have been identified [25,27,28,29]. Microcystin-LR (MC-LR)—where variable L-amino acids include leucine (L) and arginine (R) in positions (2) and (4), respectively—is one of the most common and toxic congeners, with i.p. LD_50_ values ranging from 25 to 125 µg kg^−1^ b.w. in mice [13,25,29,30]. According to WHO recommendations, the provisional drinking water guideline values of MC-LR are 1 µg L^−1^ [29,30,31]. A water body is considered dangerous for recreational purposes when the number of cyanobacteria reaches 20 × 10^3^ cells mL^−1^, and the MC concentrations exceed 2–4 µg L^−1^ [30].

MCs are synthesised in the nonribosomal pathway via a multifunctional modular enzyme complex consisting of a combination of nonribosomal peptide synthetases (NRPS), type I polyketide synthases (PKS-I) and tailoring enzymes [32,33,34,35]. The microcystin gene cluster (*mcy*) composed of nine to ten genes was determined for species of the genera *Microcystis*, *Anabaena*/*Dolichospermum*, *Planktothrix*, *Nostoc*, *Fischerella* and *Phormidium* enzymes [32,33,34,35,36,37,38,39,40,41].

Although the chemical structure and molecular basis of MCs production have been determined in detail, their biological role is still unknown [42,43]. The synthesis of secondary metabolites, including toxins, is associated with high metabolic costs. Gene encoding MCs are ancient and occupy a significant part of the cyanobacterial genome, which testifies to some of the advantages of their production for cyanobacteria, most likely including a competitive advantage [4,43,44].

Eutrophication and global climate change are usually cited as the main drivers of cyanobacterial blooms and the mass development of toxin-producing species throughout the world [3,4,5,7,8,27,45,46,47]. The anthropogenic eutrophication of water bodies—an increase in the nutrient load caused by human activity—is a rapid process in contrast to slow natural eutrophication lasting thousands and tens of thousands of years. The negative consequences of anthropogenic eutrophication in water bodies can manifest as an “environmental disaster” within several years or decades. The key factors of anthropogenic eutrophication include: an influx of mineral and organic substances from the atmosphere polluted by industrial activities, hydraulic engineering and surface runoff from agricultural land and urban areas as well as domestic and industrial wastewater.

Toxic bloom events are especially relevant in urbanised regions with developed industries, as well as in countries with a warm climate and scarce drinking water. Toxic cyanobacterial blooms have a major negative impact on the large water bodies which are used as drinking water sources and for recreation by a great number of people. The Laurentian Great Lakes are such an example: Lake Michigan [48], Lake Huron [49], Lake Ontario [50] and Lake Erie [51,52], as well as the largest freshwater lakes in China: Poyang Lake [53], Dong Ting Lake [54], Lake Taihu [11,12] and Lake Victoria in Africa [55].

**Figure 1:**
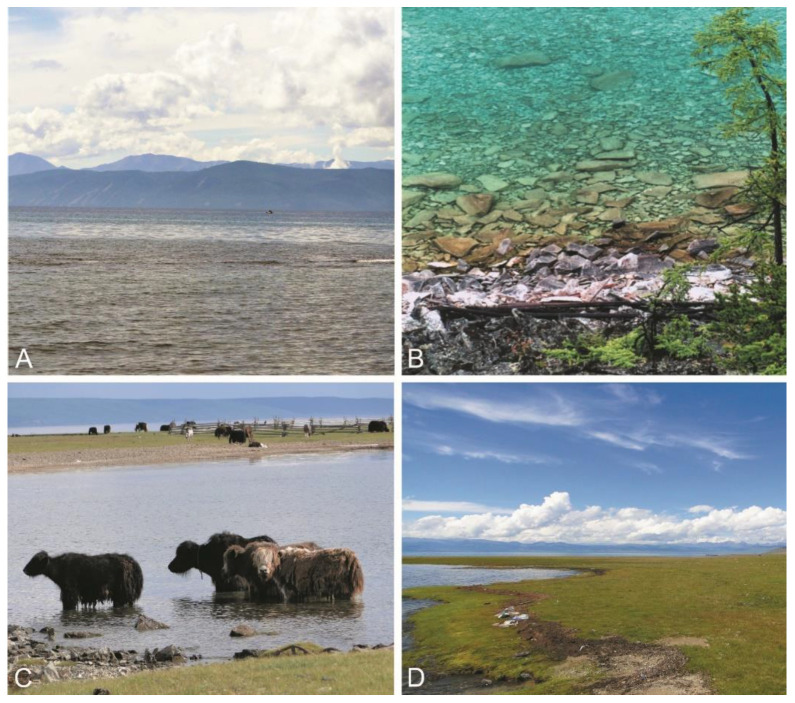
Northern part of Lake Khubsugul. (**A**) General view of the lake and the surrounding mountains on the western shore; (**B**) stony littoral of the lake; (**C**) yaks in the water and on the shore near a Khankh settlement; (**D**) plastic bottles on the shore near a Khankh settlement. Photo by T. Butina.

We aimed to revise the diversity of cyanobacteria and search for toxigenic and toxin-producing cyanobacteria in ancient oligotrophic Lake Khubsugul located in a sparsely populated highland part of Northern Mongolia. Lake Khubsugul is ranked 16th in the area among all freshwater lakes on the Earth and the second among Mongolian lakes, containing about 70% of all freshwater reserves in the country. The severe climate in the region is unfavourable for versatile agricultural and industrial activities, and nomadic pastoralism is the main occupation of the population (Figure 1).

Despite the remoteness and poor accessibility of the lake, the first data on phytoplankton in Lake Khubsugul appeared at the beginning of the 20th century (reviewed by [56]). Over a century of study, the authors emphasised the absence of cyanobacteria in the lake many times, along with predominance of diatoms and green algae [57,58,59,60,61,62,63]. Cyanobacteria were first identified in the lake in the late 1980s. Seven of the 76 species found in plankton belonged to cyanobacteria and one species was identified in benthos [64,65]. The latest list contains 97 taxa of planktonic algae, while the taxonomic composition and number of cyanobacteria remain the same [56]. The morphotypes and abundance of picoplanktonic cyanobacteria and bacteria have been determined quite recently, using electron and fluorescent microscopy [66]. Studies of the microbial diversity of aquatic ecosystems in Mongolia using high-throughput sequencing (HTS) have not yet been carried out.

In this study, we assessed the water quality based on hygiene-microbiological and hydrochemical indicators, investigated microbial communities including opportunistic and pathogenic bacteria using amplicon-based sequencing targeting the V3–V4 region of the 16S rRNA gene, detected cyanobacteria containing the microcystin synthetase gene E (*mcy*E) by PCR-based methods, identified microcystin congeners via mass spectrometry and determined MC concentration by enzyme-linked immunosorbent assay (ELISA). At the same time, we tried to carry out a comprehensive assessment of the current state of the lake and forecast the development of cyanobacterial communities in the nearest future, taking into account the obtained results and the not readily available literature data from previous decades, as well as the literature data on the evaluation of global climate change in Mongolia.

## 2. Results

### 2.1. Hydrophysical and Hydrochemical Parameters

From 11 to 14 July, the surface water temperature in the northern part of Lake Khubsugul ranged from 8.9 °C to 9.5 °C. Mean water transparency measured by Secchi disk was 20 m (SD = 0.5) in the pelagic zone. The pH value in the surface water layer was 8.4 to 8.5 at all stations, slightly decreasing with depth (to pH = 8.1 at a depth of 25 m).

Hydrophysical and hydrochemical parameters at different depths and in the 0–25 m layer are presented in Table 1. The water in the lake was well aerated throughout the water column; the oxygen saturation reached 100 to 105%. The concentrations of nutrients in the lake water were low and similar at all the stations. Silica (Si) and nitrate ion (NO_3_^–^) were rather evenly distributed in the upper 25 m layer. Nitrite (NO_2_^−^) and ammonium (NH_4_^+^) ions were detected only in the surface layer at trace amounts. The concentration of inorganic phosphorus (PO_4_^−^) changed insignificantly with depth and was 2.3 to 2.7 µg L^−1^. The ratio of the total nitrogen to total phosphorus (TN/TP) in the lake water averaged 32, indicating a phosphorus deficiency and high probability of limiting plankton growth. The maximum concentrations of organic nitrogen (N_org_) and phosphorus (P_org_) was determined at a depth of 15 m. The chemical oxygen indices (COD_Mn_ and COD_Cr_) in the pelagic zone of Lake Khubsugul were low during the study period and their ratio (100 * COD_Mn_/COD_Cr_) averaged approximately 19%, indicating the predominance of easily hydrolysable organic matter in the water. The concentration of organic carbon in the water of Lake Khubsugul on average was 1.0 mg C L^−1^.

### 2.2. Chlorophyll a

In July, the concentration of chlorophyll *a* (Chl *a*) in the plankton of Lake Khubsugul was low (Table 1), which is typical of oligotrophic waters [67]. The data on the Chl *a* concentration in biofilms covering the surface of stones in Lake Khubsugul varied significantly depending on the sample type. Chl *a* content in cyanobacterial biofilms averaged 324 (SD = 97) µg g^−1^ wet weight; it was higher in biofilms dominated by green algae.

**Table 1:** Hydrophysical and hydrochemical parameters in the pelagic zone of Lake Khubsugul.

Components/ Parameters	Units	Depth, m											
		0		5		10		15		25		0–25	
		Mean	SD	Mean	SD	Mean	SD	Mean	SD	Mean	SD	Mean	SD
Ec (25 °C)	µS cm^−1^	171.4	1.0	182.4	2.2	179.1	1.96	182.8	1.5	133.9	1.4	169.9	1.6
Temperature	°C	9.1	0.06	8.4	0.1	8.4	0.15	8.0	0.1	8.0	0.1	8.38	0.1
Oxygen	mg L^−1^	10.98	0.04	11.31	0.03	11.39	0.04	11.42	0.02	11.75	0.03	11.37	0.03
Si	mg L^−1^	0.82	0.02	0.82	0.01	0.81	0.01	0.81	0.02	0.77	0.02	0.80	0.02
N-NH_4_	mg L^−1^	0.010	0.001	<0.01		<0.01		<0.01		<0.01			
N-NO_2_	mg L^−1^	0.001	0.0006	<0.001		<0.001		<0.001		<0.001			
N-NO_3_	mg L^−1^	0.010	0.001	0.010	0.001	0.012	0.002	0.010	0.001	0.014	0.001	0.11	0.001
DIN ^1^*	mg L^−1^	0.021	0.0015	0.010	0.001	0.012	0.002	0.010	0.001	0.014	0.001	0.014	0.001
N_org_ *	mg L^−1^	0.07	0.01	0.08	0.01	0.09	0.01	0.13	0.02	0.10	0.01	0.9	0.01
TN	mg L^−1^	0.09	0.01	0.09	0.01	0.10	0.01	0.14	0.02	0.11	0.01	0.11	0.01
DIP ^2^	µg L^−1^	2.3	0.3	1.6	0.5	2.0	0.2	1.6	0.4	2.7	0.6	2.0	0.4
P_org_ *	µg L^−1^	0.4	0.09	1.1	0.4	2.3	0.2	2.7	0.4	0.6	0.1	1.42	0.2
TP	µg L^−1^	2.7	0.3	2.7	0.4	4.3	0.1	4.3	0.2	3.3	0.1	3.5	0.23
COD_Cr_	mgC L^−1^	1.01	0.07	0.95	0.03	1.55	0.12	0.51	0.09	1.23	0.08	1.05	0.08
COD_Mn_	mgC L^−1^	0.15	0.02	0.15	0.03	0.16	0.02	0.21	0.04	0.16	0.02	0.17	0.02
Chlorophyll *a*	µg L^−1^	0.20	0.02	0.42	0.07	0.54	0.05	0.55	0.15	0.53	0.13	0.45	0.08

^1^ DIN—dissolved inorganic nitrogen; ^2^ DIP—dissolved inorganic phosphorus; *—calculated values.

### 2.3. Microscopy Analysis of Planktonic and Benthic Cyanobacterial Communities

**Figure 2:**
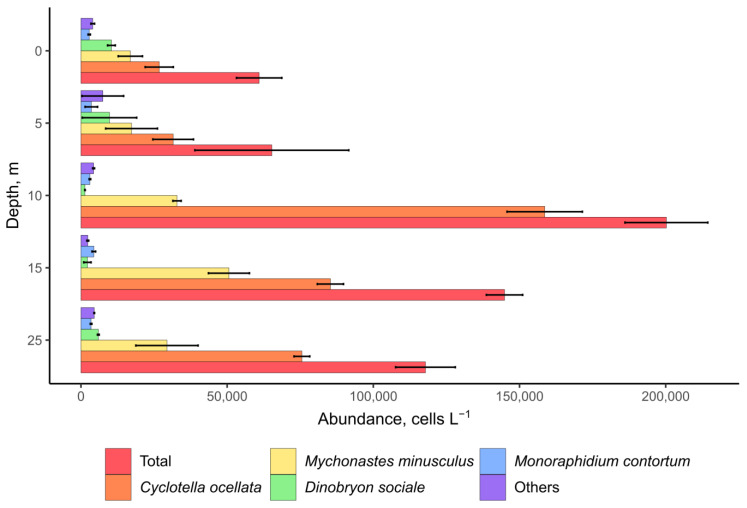
Vertical distribution of mean phytoplankton abundance, including the contribution of dominant species at discrete depth intervals from 0–25 m for pelagic stations 1N, 11–13Kh. Horizontal error bars representing the standard deviation are included.

**Figure 3:**
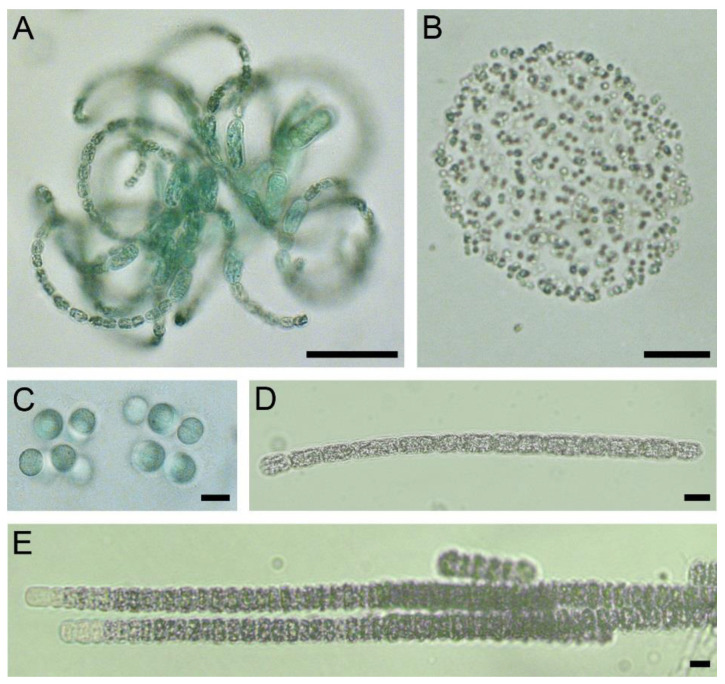
Planktonic cyanobacteria in Lake Khubsugul. Light microscopy images. (**A**) *Dolichospermum lemmermannii*; (**B**) *Coelosphaerium kuetzingianum*; (**C**) *Limnococcus limneticus*; (**D**) *Planktothrix* sp.; (**E**) *Trichodesmium lacustre*. Scale bar (**A**,**B**) = 50 μm; (**C**–**E**) = 10 μm.

Microscopy analysis of plankton samples from Lake Khubsugul indicated that the diatom *Cyclotella ocellata* dominated phytoplankton; its abundance reached 170 thousand cells L^−1^, and its contribution to the total abundance of phytoplankton was 80% (Figure 2). Chrysophycean algae *Dinobryon sociale* and green algae *Mychonastes minusculus* and *Monoraphidium contortum* were subdominant; other algae were minor. The autotrophic picoplankton was numerous and was mainly represented by cells belonging to the morphotypes of the genera *Synechococcus* and *Cyanobium*. *Dolichospermum lemmermannii* and *Limnococcus limneticus* represented nanoplanktonic cyanobacteria with an abundance of up to 689 cells L^−1^ and 70 cells L^−1^, respectively (Figure 3A,C). The total abundance of nanoplanktonic algae and cyanobacteria reached 200 thousand cells L^−1^ (Figure 2). The cyanobacterial species, *Coelosphaerium kuetzingianum*, *Planktothrix* sp. and *Trichodesmium lacustre* were found in net samples (Figure 3B,D,E).

**Figure 4:**
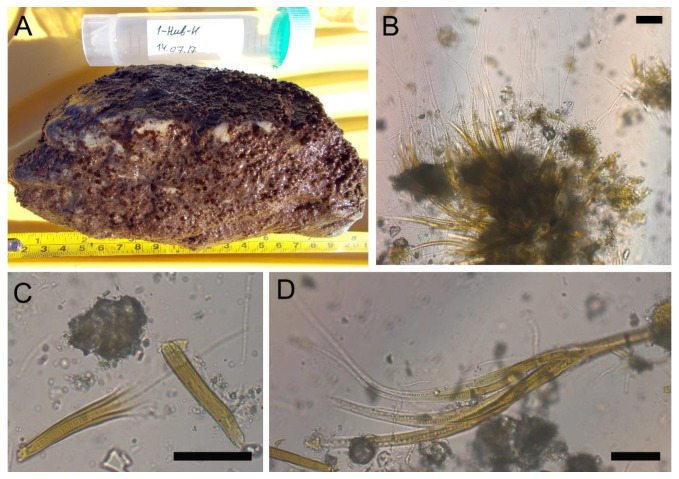
Benthic cyanobacteria in Lake Khubsugul. (**A**) General view of the stone with cyanobacterial biofouling; (**B**–**D**) cyanobacterial biofouling under light microscope, scale bar = 50 μm; (**B**) a colony of the *Rivularia coadunata* cyanobacteria with calcium carbonate encrustations (dark spots); (**C**) single filament of *R. coadunata* with a funnel-shaped extension of sheath; (**D**) branching filament of *R*. *coadunata*.

Examination of benthic samples revealed two types of foulings on the stony substrates. *Ulothrix zonata* dominated the samples, with all-over green foulings from the northern margin of the lake. The diatom *Hannaea arcus* and cyanobacteria *Leibleinia epiphytica* were epiphytes on the filaments of *U. zonata*; cyanobacteria *Calothrix parietina* was found among the filaments. The second type of foulings identified near the Khankh settlement was in the form of separate or confluent small hemispherical brown colonies (Figure 4A). Diatoms and cyanobacteria of the orders Synechococcales, Chroococcales and Nostocales dominated by *Rivularia coadunata* prevailed in their composition (Figure 4B–D). The number of *R*. *coadunata* reached 58 million filaments m^−2^, and the biomass—4458 g m^−2^. *Nostoc* sp. with the average biomass of 160 g m^−2^ was present in benthos (Table S1).

Foulings on biogenic substrates were mainly dead filamentous green algae, dominated by six species of cyanobacteria: *Calothrix parietina*, *Nostoc* sp., *Leibleinia epiphytica*, *Chamaesiphon subglobosus*, *Chamaesiphon polonicus*, *Chroococcus* sp. and *Pseudanabaena galeata* (Table 2). The total number of benthic cyanobacteria on the stones averaged 82 million ind. m^−2^ (Table S1). Moss thickets were seen on the sandy bottom at the northern margin of the lake, while cyanobacterial biofilms and other foulings were absent.

### 2.4. High-Throughput Sequencing of 16S rRNA Gene of Bacterial Community

**Figure 5:**
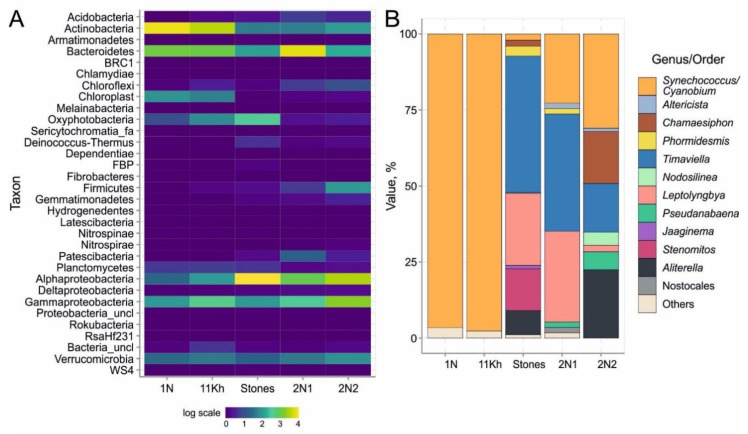
Taxonomic structure of plankton (1N, 11Kh) and benthic biofilm (“Stones”, 2N1, 2N2) communities in Lake Khubsugul: (**A**) heat map diagram with bacteria and chloroplast OTUs. Values are presented on a logarithmic scale; (**B**) cyanobacteria ratio. Minor OTUs (less than 1% of any sample) and unclassified sequences are defined as “others”.

In plankton and benthic biofilms, we obtained 206,809 sequences of 16S rRNA gene fragment with an average read length of 450 bp. Figure S1 shows the number of obtained sequences, identified OTUs and diversity indices (Chao1, ACE, Shannon and Simpson) calculated for all OTUs, except for singletons and duos (Figure S1). Sequences belonging to the phyla Actinobacteria (38–53%), Bacteroidetes (22–23%), Proteobacteria (10–22%), Cyanobacteria (1.8–5.9%), Verrucomicrobia (3.3–4.4%) and Planctomycetes (1.0–1.2%) prevailed, and the algal chloroplast 16S rRNA sequences were also numerous (4.8–6.5%) (Figure 5A). The minor phyla included Chloroflexi, Firmicutes, Armatimonadetes, Chlamydia, Chlorobi, Acidobacteria, Deinococcus-Thermus, Gemmatimonadetes, Patescibacteria and Nitrospirae. Planktonic cyanobacteria were mainly presented by the genera *Cyanobium*/*Synechococcus* (family Cyanobiaceae, order Synechococcales): their proportion was 21 to 54% of all Oxyphotobacteria sequences (Figure 5B).

Proteobacteria (33–68%) were dominant in biofilms, among which Alphaproteobacteria (20–61%) prevailed, and Bacteroidetes (7.8–50%), Actinobacteria (4.7–7.2%) and Verrucomicrobia (2.5–6.3%) were common. In two benthic samples, the proportion of Chloroflexi (1.1–1.9%) and Firmicutes (1.1–7.2%) was high, while the proportion of cyanobacteria was low (1%).

Contrarily, the contribution of cyanobacteria increased to 13% in the green biofilm sample (Figure 5A). OTUs of filamentous and coccoid cyanobacteria of the order Synechococcales were the most numerous: up to 87% and 47%, respectively (Figure 5B). The phylotypes of *Synechococcus* (99% identity with *Synechococcus* sp. MW6C6) dominated the coccoid forms. The diversity of phylotypes was almost two times higher than the number of species based on microscopy data (Table 2). The greatest diversity of cyanobacterial OTUs (17) was observed in the samples taken from stones near the Khankh settlement. We detected eight cryptic genera of Synechococcales with similar morphology: thin filamentous cyanobacteria of the phylotypes *Jaaginema*, *Leptolyngbya*, *Nodosilinea*, *Phormidesmis*, *Pseudanabaena*, *Shackletoneilla*, *Stenomitos* and *Timaviella*. Their contribution to the total number of cyanobacterial phylotypes ranged from 28 to 87% (Figure 5B). We identified Nostocales cyanobacterial phylotypes of the genera *Calothrix*, *Nostoc*, *Rivularia* and *Scytonematopsis*, which amounted to less than 2% of the total number of cyanobacterial phylotypes. For the first time, we found a rare member of the order Gloeobacterales, the oldest branch of cyanobacteria without thylakoids, the phylotype *Gloeobacter kilaueensis*.

### 2.5. Microbial Water Quality Assessment

Water quality in all samples from the lake corresponded to the sanitary and hygiene standards and requirements established by the sanitary rules and norms, imposed in Russia for assessing water use objects of I and II categories [68,69,70] (Table S2). The number of faecal derived bacteria and phages as sensitive indicators of recent faecal contamination was significantly below the guideline values. We detected total coliforms in six coastal and two pelagic samples, ranging from 2 ± 1 to 54 ± 2 colony-forming units (CFU) in 100 mL (with a safety standard ≤ 1000 and 500 CFU in 100 mL for the two categories of water use objects, respectively). Thermotolerant coliforms and *Escherichia coli* were identified in four coastal and two pelagic samples, the number of which did not exceed 4 ± 0 CFU in 100 mL (with a safety standard ≤ 100 CFU in 100 mL). Enterococci (bacteria of the genus *Enterococcus*) were present in five coastal samples and two pelagic samples (1–6 ± 1 CFU in 100 mL, with a safety standard of ≤ 100 and 10 CFU in 100 mL for each category, respectively). In 12 samples, we did not find total coliforms, thermotolerant coliforms and *E. coli*, eight of which were pelagic samples. No coliphages were detected in the water from Lake Khubsugul. The coastal stations near the Khankh settlement had higher values of total and thermotolerant coliforms compared to the northern stations.

### 2.6. High-Throughput Sequencing of 16S rRNA Gene of Pathogenic and Opportunistic Bacteria

**Figure 6:**
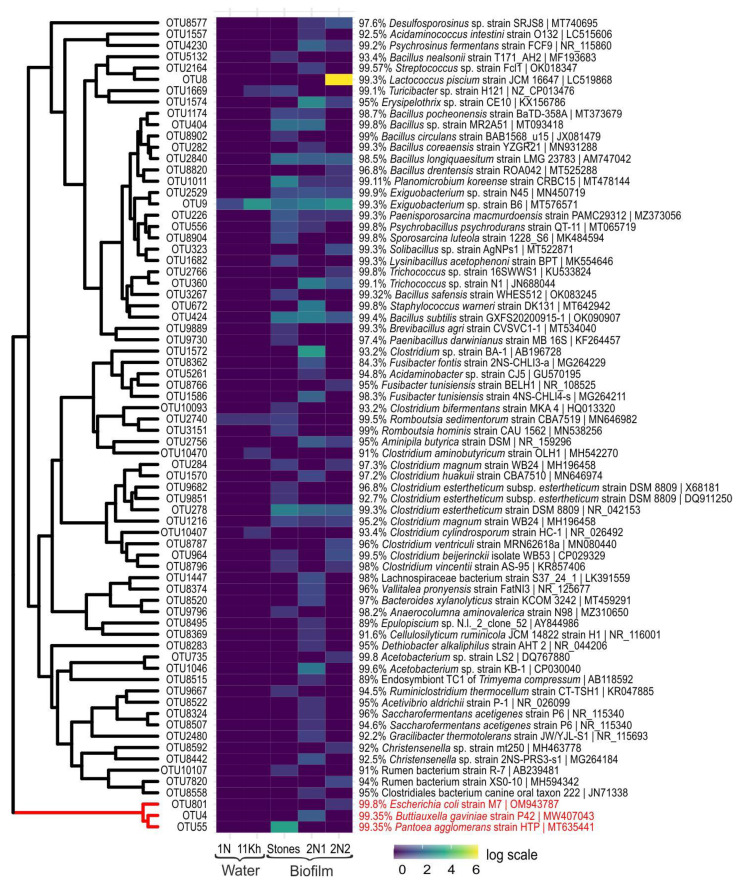
Heat map diagram with Firmicutes (black colour) and Enterobacteriaceae family (Proteobacteria) (red colour) OTUs in Lake Khubsugul samples. Values are presented on a logarithmic scale.

Analysis of microbial communities using HTS revealed the presence of pathogenic and opportunistic bacteria of the families Enterobacteriaceae, Moraxellaceae, Legionellaceae and Vibrionaceae (class/phylum Gammaproteobacteria) in the water and in biofilms. Enterobacteriaceae that include the greatest number of bacteria harmful to human health were not found in the water; they were present in biofilms in small amounts. The biofilms contained sequences identified as *Escherichia coli* (99.8%), a well-known member of the human and animal gut microbiome, and *Pantoea agglomerans* (99.3%), the most commonly isolated species in humans [71] (Figure 6). We detected sequences similar to the widely distributed species *Buttiauxella gaviniae* (99.3%) isolated from molluscs, mainly snails and slugs, which is possibly pathogenic to humans [72].

The bacteria of the genus *Acinetobacter* (Moraxellaceae) were the most numerous; the water and biofilms contained *Acinetobacter nosocomialis* (99.3%), *A. radioresistens* (99.5%) and *A. lwoffii* (99%). *Acinetobacter* spp. are ubiquitous inhabitants of water; however, they are opportunistic pathogens that may cause urinary tract infections, pneumonia, bacteraemia, secondary meningitis and wound infections, predominantly in immunosuppressed persons [31].

The biotopes included a negligible amount of phylotypes of the genus *Legionella* (Legionellaceae) causing acute infectious diseases, among which are *L. feeleii* (96%), *L. quateirensis* (97.8%), *L. nautarum* (97.8%), *L. birminghamensis* (95.2%), *L. anisa* (96.1%), *L. saoudiensis* (96.3%), *L. taurinensis* (93%) and *L. rowbothamii* (97.8%). In biofilms, there were sequences of *Vibrio fluvialis* (Vibrionaceae) (99.1%), bacteria transported by water, which was first isolated in the 1970s in patients with severe diarrhoea in Bahrain [73].

The sequences of the phylum Firmicutes were mainly present in the coastal water; bacteria of the order Lactobacillales predominated among them being largely represented by the species *Lactococcus piscium* (99.3%) isolated from diseased rainbow trout before [74]. Notably, bacteria associated with fish were found in the studied samples, for example, *Epulopiscium* sp., gram-positive bacteria that had a symbiotic relationship with surgeonfish [75].

We determined sequences closely related to *Streptococcus* sp. (99.5%) inhabiting the human respiratory and digestive tracts. Bacteria of the order Bacillales were numerous and diverse; they had more than 97% identification with bacteria from other habitats. Representatives of the genus *Bacillus* turned out to be mainly soil species, while some of them were the inhabitants of the gastrointestinal tract of ruminants and humans, for example *B. subtilis* [76]. In the samples, we identified the sequences of *Bacillus circulans*, described as a human pathogen associated with “sepsis, mixed infections and wound infections”, as well as with meningitis. Most members of *Bacillus* species are distributed in natural environments, but some of these species are able to cause severe to self-limited disorders as an actual or opportunistic pathogen, such as *B. circulans* [77]. Among staphylococci, there was *Staphylococcus warneri* (99.8%), a member of normal microflora of the skin and mucous membrane of some organs in humans, primates and domestic animals [78].

Among Firmicutes, there were numerous bacteria of the order Clostridiales in the water and biofilms. Most species of this order are saprophytes inhabiting many environments, primarily the soil. They are found in healthy people and comprise a substantial part (several tens of percent) of the normal microflora in the oral cavity, intestines and human urogenital system. Among the sequences that were 98 to 99% identical to the representatives of the human microflora, there were bacterial genera belonging to the family Christensenellaceae, which inhabit the human gut and are important for human health [79].

The genus *Clostridium* was diverse, accounting for over 10 OTUs. Bacteria belonging to the genus *Clostridium* are anaerobic Gram-positive rods with the ability to form endospores [80]. *Clostridium* species are ubiquitous in the environment. *Clostridium* species are a part of the normal flora of the intestinal tract of humans and other animals, and may also be isolated from the female genital tract and the oral mucosa [80]. In our case, we detected potentially pathogenic *Clostridium beijerinckii* and *Clostridium ventriculi* (formerly *Sarcina ventriculi*), as well as *Cellulosilyticum ruminicola* isolated from the rumen of a yak in Mongolia [81].

*Romboutsia hominis* (99.5%) [82], *Turicibacter* sp. H121 (99.1%) [80] and *Acidaminococcus intestini* (99%) [83,84] species are considered useful markers of anthropogenic impact; bacteria that are commonly detected in the gastrointestinal tracts and faeces of humans and animals.

### 2.7. Microcystin Synthetase Genes

PCR analysis yielded a positive result for net phytoplankton samples collected in the coastal zone of Lake Khubsugul near the Khankh settlement. No cyanobacteria with MC synthetase genes were found in the plankton from pelagic and benthic samples.

We obtained 30 sequences from PCR positive samples representing three different genotypes. Three sequences of the *mcy*E gene had the greatest identification (98.3%) with the sequences of the genus *Microcystis*, in particular, with the strain *Microcystis aeruginosa* NIES-88, isolated from Lake Kawaguchi (Yamanashi, Japan). Fifteen sequences were closely related (99.6%) to the sequences of uncultured cyanobacteria isolated from Lake Baikal biofilms [85] and were 92% identical to the *Snowella* sp. CHAB strains 6601, 6602, 6604, 6605 and 6606, isolated from a brackish water lake in Qinghai-Tibetan Plateau (China) (unpublished data). Cultured strains of *Nostoc*, for example, *Nostoc* sp. 152 (93%) and *Nodularia spumigena* UHCC0039, CCY9414, NSOR10 (91.1–91.5%) were the closest relatives of 12 sequences.

The phylogenetic tree showed amino acid sequences of the *mcy*E gene from Lake Khubsugul, forming stable clusters with closely related sequences (Figure S2). It was previously shown that branching of *mcy*E genes corresponds to highly supported diversification according to *Microcystis*, *Anabaena*, *Nodularia*, *Planktothrix*, *Nostoc* and *Phormidium* genera [86].

Within the genus *Microcystis* (order Chroococcales) cluster, genotypes from Lake Khubsugul were grouped together with the sequences of *M*. *aeruginosa*, *M. flos-aquae*, *M*. *viridis* and *M*. *wesenbergii*, mainly from the Asian water bodies, including Lake Baikal. Amino acid sequences of the order Nostocales from Lake Khubsugul formed a stable cluster with *Nostoc* sp. “*Peltigera membranacea* cyanobiont” 232 within the clade formed by clusters of nodularin-producing *Nodularia spumigena* and microcystin-producing *Nostoc* sp. 152 and *Nostoc* sp. “*Peltigera membranacea cyanobiont*” 213.

Sequences of members of the order Synechococcales from Lake Khubsugul were included in the cluster, together with those of the *Snowella* sp. CHAB strains and uncultured sequences were isolated from Lake Baikal benthos.

### 2.8. Microcystin Concentration and Microcystin Congeners

The initial screening of MCs was performed using the Microcystins-Adda ELISA kit. All samples and standards were analysed in duplicate. There were no MCs in water samples and net samples from Lake Khubsugul, in the biofilms from stones sampled in the coastal zone of the northern margin of the lake (LODs for the Adda ELISA < 0.10 ppb). We detected MCs in biofilms from stony substrates sampled in the coastal zone near the Khankh settlement (eight biofilms, station “Stones”); mean MC concentration was 41.95 µg g^−1^ d. wt. (SD = 5.57) (Figure S3).

Integrated samples of eight biofilms of stone substrates from the coastal zone off the Khankh settlement contained five MC congeners, according to the high performance liquid chromatography-high resolution mass spectrometry results (HPLC-HRMS/TOF): MC-YR, MC-LAba, MC-LY, [ADMAdda^5^]MC-LR and [ADMAdda^5^]MC-XR (where X is a fragment, its structure has not yet been determined) (Figure S4). The content of these MCs was estimated using a calibration curve for MC-LR standard solution (Biosense Laboratories, Norway), as follows: 50 (SD = 7), 0.4, 0.2, 0.7 and 4.5 (SD = 1.4) μg g^−1^ d. wt., respectively.

### 2.9. Statistical Analysis

**Figure 7:**
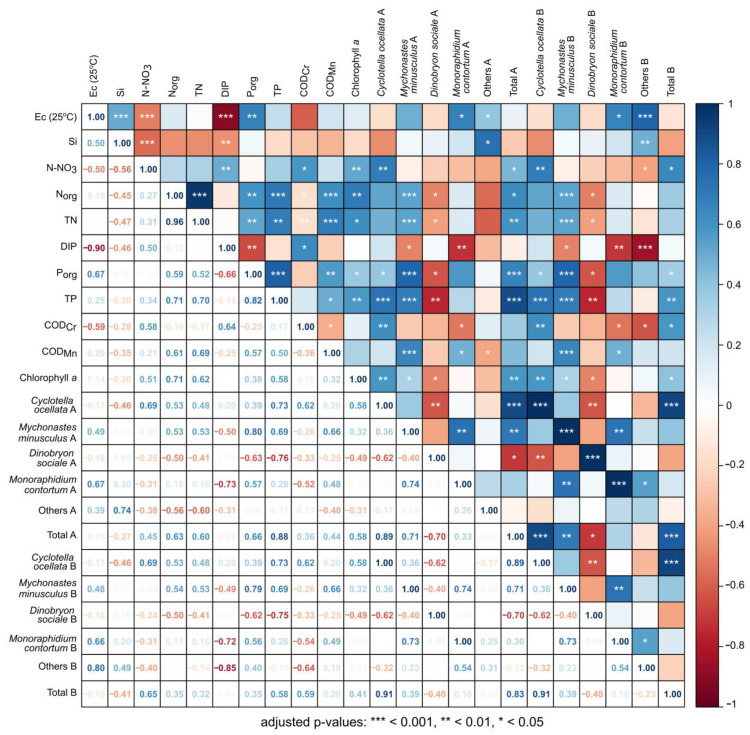
Heat map of correlation analysis among chemical composition in water samples, based on Spearman’s rank correlation matrix. Adjusted *p*-values are shown in the upper triangular of the correlation matrix with asterisks. The bottom triangular contains Spearman’s coefficients. The colour intensity is proportional to the correlation coefficient. Abbreviations: A—abundance, B—biomass.

According to Spearman’s rank correlation matrix, there were strong positive correlations between total phytoplankton abundance and the chlorophyll *a* concentration (*r* = 0.58, *p* < 0.01); phytoplankton abundance and dominant species such as diatom *Cyclotella ocellata* (*r* = 0.89, *p* < 0.001) and green algae *Mychonastes minusculus* (*r* = 0.71, *p* < 0.01) numbers were revealed (Figure 7).

The phytoplankton number was positively related to the following hydrochemical parameters: the concentrations of total (*r* = 0.6, *p* < 0.01) and organic nitrogen (*r* = 0.63, *p* < 0.05), total (*r* = 0.88, *p* < 0.001) and organic phosphorus (*r* = 0.66, *p* < 0.001).

Nitrate nitrogen concentration (NO_3_^–^) was positively correlated with total phytoplankton abundance (*r* = 0.45, *p* < 0.05) and biomass (*r* = 0.65, *p* < 0.05), *C. ocellata* abundance (*r* = 0.69, *p* < 0.01) and biomass (*r* = 0.69, *p* < 0.01), chlorophyll *a* concentration (*r* = 0.51, *p* < 0.01). The established relationships indicated that algae accumulated nitrate nitrogen during the observation period, and its concentration was sufficient to increase phytoplankton biomass.

A weak positive correlation was noted between dissolved inorganic phosphorus (DIP) and *C. ocellata* number. Strong negative correlations were indicated between DIP and green algae *Mychonastes minusculus* (*r* = −0.49, *p* < 0.05), *Monoraphidium contortum* (*r* = −0.72, *p* < 0.01) and other algae (*r* = −0.85, *p* < 0.001) biomasses. It seems likely that dissolved inorganic phosphorus limited the growth of these algae groups. The DIP concentration in the water of the lake was low; organic phosphorus prevailed over inorganic phosphorus at the depths of 10–15 m, indicating the active production of organic matter in these horizons. Based on the data obtained, it can be concluded that the water of Lake Khubsugul was characterised by the lack of inorganic phosphorus for phytoplankton growth.

Chrysophycean algae *Dinobryon sociale* should be specially mentioned: its abundance was significantly negatively correlated with chlorophyll *a* (r = −0,49, *p* < 0,05), organic phosphorus (*r* = −0.63, *p* < 0.05) and total phosphorus (*r* = −0.76, *p* < 0.01), as well as organic (*r* = −0.50, *p* < 0.05) and total (*r* = −0.41, *p* < 0.05) nitrogen concentration. *D. sociale* is a mixotrophic species, it probably leads a phagotrophic lifestyle in Lake Khubsugul, thus depending on organic nitrogen and phosphorus.

Easily oxidised organic compounds (COD_Mn_) significantly positively correlated with total (*r* = 0.69, *p* < 0.001) and organic nitrogen (*r* = 0.61, *p* < 0.001), with organic (*r* = 0.57, *p* < 0.01) and total phosphorus (*r* = 0.50, *p* < 0.05), indicating that nitrogen compounds predominated in the lake.

**Figure 8:**
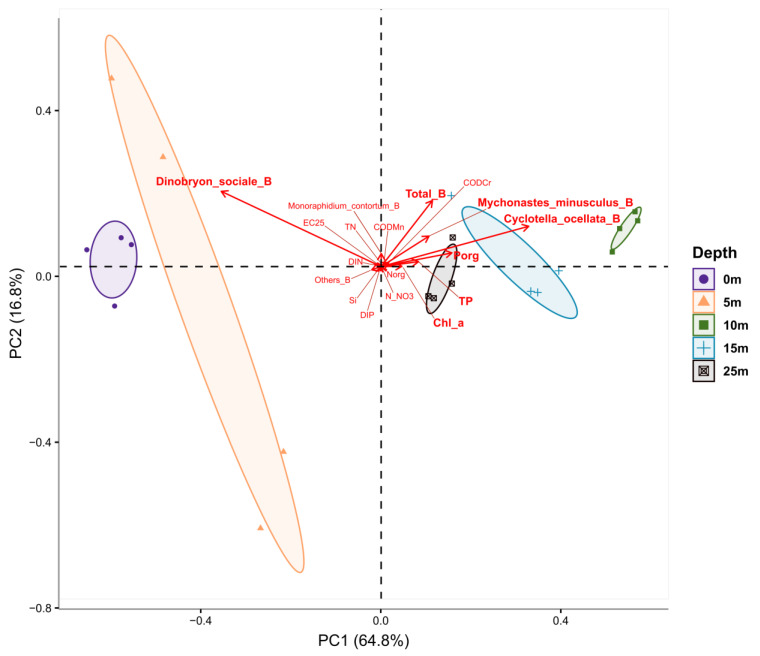
PCA biplot was based on a log10 transformed data matrix of nutrients concentrations. The biplot shows the PCA scores of the explanatory variables as vectors (in red) and parameters (i.e., nutrients concentration) at each depth on the first (*x*-axis) and second (*y*-axis) PCs. The location of parameters on the same side as a given variable means that they had a high contribution to this variable. The magnitude of the vectors (lines) represents the strength of their contribution to each PC. Vectors pointing in similar directions indicate positively correlated variables, vectors pointing in opposite directions indicate negatively correlated variables and vectors located approximately at right angles to each other indicate low or no correlation. Coloured ellipses of concentration (size determined by the 0.95 probability level) reflect observations clustered by depth.

Most of the biological and hydrochemical parameters were significantly distinct by depth after non-parametric Kruskal–Wallis (KW) test. Boxplots for each factor are arranged in Figure S5 and statistics for KW test are presented in Table S4. According to the implemented Principal Component Analysis (PCA), most of the variations in the data were covered by the two Principal Components (PCs)—81.6%: 1st Principal Component explained 64.8% of the variance and the 2nd Principal Component—16.8% (Figure 8). As shown on the PCA graph, the greatest variability at depths from 0 to 5 m was marked for “*Dinobryon_sociale*_B”. At depths from 10 to 25 m, “Total_B”, “*Mychonastes_minusculus*_B”, “*Cyclotella_ocellata*_B”, “P_org_”, “TP” and “Chl_a” had the highest variability. Moreover, the last three indicators had a strong positive correlation, because their vectors were oriented in the same direction and had a rather small angle in relation to each other.

The other indicators, such as “TN”, “Ec_25C”, “Si”, etc. had reliable differences in depth according to the KW test, but due to their low variability, made minimal contribution to the overall distribution.

## 3. Discussion

For the first time, we detected cyanobacteria that contain genes encoding microcystin synthetase and produce microcystins in the high-altitude, deep, ancient Lake Khubsugul, which is located in a region with a severe climate and widespread permafrost. The diversity and abundance of planktonic cyanobacteria in the lake is extremely low. Benthic cyanobacteria are richer in species composition and are more abundant on stone substrates. Our measurements of the water transparency, concentration of nutrients, total nitrogen and phosphorus and chlorophyll *a* in Lake Khubsugul were consistent with the parameters typical of oligotrophic water bodies [67]. Based on the concentration of organic matter, the water in Lake Khubsugul can be classified as “extremely clean” [87].

In the 1970s to 1990s, extensive information about chemical composition of Lake Khubsugul water was obtained as a result of joint Russian–Mongolian [57,88,89,90] and other international expeditions [91] that served as a basis for assessing the changes that had been occurring in recent decades. In 2017, the concentrations of mineral and organic phosphorus and organic carbon in Lake Khubsugul were almost the same as the values determined previously [57,88,89,90,91].

We registered a silica concentration drop in Lake Khubsugul compared to recent decades, which was likely associated with a prolonged low water level in the region. For example, Lake Baikal has recently shown a reduction in the river runoff and a decrease in silica concentration [92]. The water contained insignificant amounts of ammonium and nitrite ions that had not been found in the 1960s to 1990s. The appearance of these inorganic nitrogen compounds—which were first detected in 2011, according to [89]—was due to permafrost melting and/or livestock overgrazing. The concentrations of nitrates, total nitrogen and chlorophyll *a* during our survey were slightly higher than in the previous years [57,66,89,91]. Water pollution with ammonium ions was detected in the coastal zone of the lake because livestock waste was transferred to the lake by rainwater, as shown in recent studies [93]. At the same time, concentrations of nitrite ion and organic nitrogen in Lake Khubsugul were lower than those in other large oligotrophic water bodies, e.g., in Lake Baikal [94,95].

The established relationships and PCA analysis indicated that phytoplankton biomass and production in the euphotic layer of water was probably limited by inorganic phosphorus; inorganic nitrogen concentration was sufficient to increase phytoplankton biomass during the study period. We still note that diatom *Cyclotella ocellata* and two species of green algae, especially *Mychonastes minusculus*, were the main phytoplankton of Lake Khubsugul. Green and diatom algae contributed most to the primary production of the lake; their amount was strictly positively correlated with chlorophyll concentration, the concentrations of total and organic nitrogen and phosphorus. A PCA analysis clearly demonstrated a non-uniform vertical distribution of phytoplankton; the diatoms and green algae preferred 10–25 m depth, where their abundance was strongly positively correlated with the main hydrochemical parameters. In the upper water column (0–5 m), the mixotrophic Chrysophycean algae *Dinobryon sociale*, not previously reported as an important phytoplankton species, appeared as a key species negatively correlated with chlorophyll *a*, organic and total phosphorus, organic and total nitrogen concentration.

Based on the data obtained, we can state that the ecosystem of Lake Khubsugul currently does not show signs of eutrophication; hydrophysical and hydrochemical parameters are unfavourable for the mass growth of cyanobacteria. We can also conclude that the trophic state of the lake has not changed over the past 50 years.

In recent decades, tourism has begun to actively develop in the Khubsugul region: recreation centres, tourist camps and holiday homes were constructed on the lake shores. People use the water from Lake Khubsugul for domestic purposes, recreation and drinking. Due to the lack of centralised sewage treatment systems, household wastewater enters the lake with rains or often directly from rustic toilets. However, the number of faecal indicator microorganisms in all studied samples did not exceed the guideline values for these two water use categories [68,69].

According to Kirschner’s surface water hygiene classification, the water in Lake Khubsugul in 2017 to 2019 corresponded to “pollution level I—low”, i.e., the number of total coliforms was ≤500 in 100 mL, and the number of faecal coliforms was ≤100 in 100 mL of water [93]. Slightly higher coliform counts compared to our values were determined in summer in the lake water near the southern and northern settlements and along the west coast of the lake, but they did not exceed the values typical of low pollution level [93]. The authors indicate that the improvement of sanitation and hygiene conditions in the tourist destinations along Lake Khubsugul contributed to the reduction of the *E. coli* and coliforms in the lake water. Therefore, the results of the microbiological water quality assessment testified to a low degree of faecal pollution of the water in Lake Khubsugul; in other words, the water quality in the lake can be described as near-pristine.

Our data suggest that faecal microorganisms reach the water body not only with household wastes, but also with the faeces of horses and large and small cattle grazing in large numbers on the lake shore. The results of high-throughput sequencing of the water and biofilm microbiomes indicated the presence of bacteria from the microbiota of the skin and gastrointestinal tract of both humans and animals, including fish (gut microbiome). Along with a low human population density in the Khubsugul region, a high number of animals was recorded: there are ten animals per one Mongolian citizen, and it is very likely that bacterial pollution is generally animal derived. Pastoralism occupies a major position in the agriculture of Mongolia. Previously, the number of livestock in the Khubsugul region was the highest in the country owing to the aimak water reserves; it amounted to 1.6 million animals, which was 7.1% of the total livestock in the country [57,88].

Despite excessive livestock grazing and tourism development, Lake Khubsugul does not experience significant anthropogenic load, partly due to adequately managed economic activities on its shores. In 1992, the lake and the adjacent territory were included in Khuvsgul Lake National Park (KLNP) in an area of 11,800 km^2^. The KLNP supports relatively small human populations: the area around the lake (Khubsugul region) is the most sparsely populated compared to other large lakes in the world [88]. KLNP serves as one of the largest centres of ecotourism in Mongolia [96].

Based on personal data and the literature, we estimate that anthropogenic stressors on the lake ecosystem are minimal.

Researchers are increasingly reporting that, in addition to the excessive supply of nutrients from watersheds, global climate change is another important factor that contributes to the intensive proliferation of cyanobacteria, leading to surface water warming and ensuing thermal stratification [3,4,5,7,8,22,45,46,47].

Lake Khubsugul environments are evidently involved in global climate changes: warming rates in Mongolia even exceed the global average ones, while northern Mongolia is the fastest-warming region of the country [97,98]. Tree-ring-based reconstructions of heatwaves and soil moisture for the past 260 years revealed a trend toward warmer and drier conditions in inner East Asia, including Mongolia [98]. From 1963 to 2002, the mean annual air temperature near station Khatgal located at the southern end of the lake rose by 1.7 °C, specifically in winter—by 3.1 °C, in spring—by 2.1 °C, in summer—by 1.4 °C, and in autumn—by 0.9 °C [96,97,99]. From 1991 to 2017, the mean annual air temperature at station Khatgal increased by 0.80 °C and amounted to −3.7 °C, and over the past decade it rose by 0.24 °C [93]. In recent years, permafrost melting in the region has contributed to the lake area expansion [96].

We assume that present global climate change affects the ecosystem of Lake Khubsugul even more than regional anthropogenic processes, firstly caused by intensive pastoralism and secondly by human activity.

Microscopic examination and genetic analysis of photoautotrophic communities revealed, as in previous years, dominance in the composition of rare but not endemic diatoms and green algae species. For the first time over the past 20 years, we have assessed the diversity of benthic cyanobacteria in Lake Khubsugul as species-poor, including nine species and 18 phylotypes of benthic cyanobacteria. *Rivularia coadunata* and *Calothrix parietina*, potentially toxic species, predominated among them. *R*. *coadunata* is a cosmopolitan species, the strains of which can synthesise MCs [6]. MC-producing isolates of *C. parietina* were described in water bodies of Spain, Saudi Arabia, Egypt and New Zealand. Moreover, this species exhibited neurotoxic activity [6,100,101]. In our study, we did not detect the species of the genus *Tolypothrix*—including the endemic *T. mongolica* that was described previously—in the rocky littoral zone at depths of between 0.2 and 10 m [65]. Cyanobacteria belonging to the order Gloeobacterales were first identified in the benthos of Lake Khubsugul. The species composition of benthic cyanobacteria in Lake Khubsugul was similar to that in Lake Baikal in 2010 and 2011, before the deterioration of the environmental situation, when *Rivularia*, *Tolypothrix* and *Chamaesiphon* spp. prevailed in the biofilms of stony substrates [102]. In general, we found that the cyanobacterial communities in plankton and benthos of Lake Khubsugul did not demonstrate signs of uniqueness and/or endemism.

MC synthetase genes were identified only in three genera: *Microcystis*, *Nostoc* and, possibly, *Snowella*. The presence of toxigenic *Microcystis* sp. was anticipated in Lake Khubsugul: in the water bodies of Northern Mongolia, this cosmopolitan species was found in well-heated areas enriched in nutrients supplied by river and coastal runoff [103]. Species of the genus *Microcystis*, typically *M*. *aeruginosa* and *M. flos-aquae*, are the most common causative agents of algal blooms and the most well-known MC producers in highly productive water bodies throughout the world [27]. Previously, the members of the genus *Microcystis* were absent in the list of algae and cyanobacteria of Lake Khubsugul. We also did not identify the *Microcystis* species in the study period. I representatives of this genus can probably be found sporadically in the samples.

Sequences related to *Snowella* sp., the second group of toxigenic cyanobacteria in Lake Khubsugul, formed a separate cluster likely representing the order Synechococcales, which also included uncultured Baikal sequences [85] and the sequences of the *Snowella* sp. CHAB strains (China) (unpublished data). It is noteworthy that all toxigenic *Snowella*-like sequences were detected in Asia. We did not identify other registered sequences of microcystin synthetase genes that reliably belonged to the order Synechococcales, although the information about the MC production by the species of this order is available. For example, the presence of *mcy*B gene was detected in 12 strains of cyanobacteria: *Synechocystis* (three strains), *Synechococcus* (six strains) and *Romeria* (two strains), but the sequences in Genbank are missing [104].

The genus *Snowella* includes seven species of freshwater unicellular and colonial cyanobacteria, two of which (*S*. *rosea* and *S. lacustris*) were detected in Lake Khubsugul. There were no MC synthesis genes in the *Snowella* ULC335 genomes from microbial mat (Belgium) and *Snowella* Erken-D5_bin-0612 from lentic water body in Sweden. However, it should be taken into account that the sequences from Lake Khubsugul may also belong to the closely related genera of the family Coelosphaeriaceae, e.g., *Coelosphaerium kuetzingianum* living in the lake.

The *Nostoc mcy*E gene sequences from Lake Khubsugul were closely related to lichen-associated *Nostoc* strains isolated from the samples of a moss carpet at Keldur, Reykjavik, Iceland: mostly to *Nostoc* sp. 232 “*Peltigera membranacea* cyanobiont”, a *Peltigera membranacea* lichen photobiont [105] and, to a lesser extent, to *Nostoc* sp. 113 *“Peltigera membranacea* cyanobiont”. *Nostoc* sp. 113 was more identical to *Nostoc* sp. 152, a well-studied MC producer isolated from the water bloom sample in Lake Saaksjarvi, Finland [106,107].

It seems most likely that the aminotransferase domain of the microcystin-synthetase gene clusters of the *Nostoc* sequences from Lake Khubsugul belongs to symbiotic cyanobacteria. *Nostoc* is a cosmopolitan cyanobacterial genus occurring in both terrestrial and aquatic ecosystems, especially in the temperate and cold regions of the world. Strains of the genus *Nostoc* are the most common cyanobacteria in various symbioses [108]. The members of the order Nostocales and the family Nostocaceae are the most active in terms of general secondary metabolite production. They can synthesise about 26% of the total known metabolites produced by filamentous cyanobacteria [109].

Five MCs congeners were identified in the integrated sample of biofilms from Lake Khubsugul using the HPLC-HRMS/TOF equipment: MC-YR, MC-LAba, MC-LY, [ADMAdda^5^]MC-LR and [ADMAdda^5^]MC-XR. MC-YR is one of the most common MC congeners in freshwater ecosystems all over the world. It is produced—along with the MC-LAba and MC-LY congeners—by members of the genus *Microcystis*, mainly *Microcystis aeruginosa*. The toxicity of these three congeners is low [25,27].

The production of highly toxic [ADMAdda^5^]MC-LR is typical for planktonic *Nostoc* sp. 152 [107] and terrestrial *Nostoc* sp. strain IO-102-I isolated from a lichen association inhabiting rocks in Finland. In the former strain, its proportion reaches 35%, and 80% of the total MCs in the latter strain [110]. The *Nostoc* sp. strain IO-102-I also synthetises the minor [ADMAdda^5^]MC-XR that was also detected in the biofilms from Lake Khubsugul.

The [ADMAdda^5^]MC-LR congener was found in microbial mat samples of the Svalbard Archipelago in the Arctic where *Nostoc* sp. was identified as a putative toxin producer using molecular methods that targeted genes involved in microcystin production [111]. Sequences of *Nostoc* sp. clones didn’t join the cluster formed by Khubsugul isolates. Based on microscopic, genetic and mass spectroscopy analyses, we assume that *Nostoc* sp. may be one of the MC producers. The detection of MCs only in benthic samples may be associated with a greater biomass of cyanobacteria in biofilms, unlike planktonic samples, in which the biomass of cyanobacteria was low even in net samples.

The MC concentrations in biofilms of stone substrates from the coastal zone of Lake Khubsugul were low when analysed by ELISA (35.4 to 52.0 µg g^−1^ d. wt.), and by HPLC-HRMS/TOF (integrated sample, ca. 55.8 µg g^−1^ d. wt.). The total concentration was much lower than in cultured benthic species (1600 to 4100 µg g^−1^ d. wt.) [101] and in reservoirs of the Segura River basin, Murcia, Spain (300 to 3300 µg mg^−1^, mean 1600 µg g^−1^ d. wt.) [100], but higher than in cyanobacterial mats from Antarctic water bodies (1 to 16 µg g^−1^ d. wt.) [112] and in Arctic freshwater ecosystems (0.10 µg g^−1^ d. wt.) [113].

It is impossible to assess the real threat of mass development of benthic toxic cyanobacteria due to the absence of international guideline values for the MC content in their biomass [112,114].

The microcystin synthetase gene cluster is ancient. Phylogenetic reconstructions revealed that housekeeping genes, 16S rDNA and *rpo*C1 genes, as well as microcystin synthetase genes, co-evolved throughout the whole evolutionary history of this toxin [44]. The sporadic distribution of microcystin synthetase genes in modern cyanobacteria suggests that the ability to produce MCs may have been lost in more advanced cyanobacterial lineages. On the other hand, cyanobacterial strains that have not previously been suspected of producing MCs may retain the genes necessary for their synthesis. Future investigations of different extreme and poorly accessible habitats, including low-productive ecosystems, will possibly detect toxic species there, updating the knowledge about their distribution.

Lake Khubsugul is young (5.5 million years old) relative to the age of cyanobacteria, which is 3.5 billion years old. Apparently, cyanobacteria with their ancient toxin synthesis genes appeared in the area of modern Lake Khubsugul long before the appearance of the lake itself, which is also reasonable for other ancient lakes, Lake Baikal in particular. The Khubsugul and Baikal basins at an early stage of the Baikal Rift Zone formation (70 to 30 Ma) were a single Archeo-Baikal system [115,116]. The active uplift of the western side of the Khubsugul depression and the acquisition of its modern features took place between 3.3 and 2 Ma, and about 8.5 Ka, when the water level in the lake became close to the modern level [117].

Baikal is the largest, deepest and most ancient (20–30 million years old) freshwater lake on the planet and is older than Lake Khubsugul [118]. Many common features in these two geographically close lakes connected by a system of rivers, including old age and common origin, similar morphological and morphometric parameters, climatic conditions, relative isolation, etc., provoked the researchers to compare Baikal and Khubsugul biota [56,57,88,91]. The authors point out significant differences in the composition, abundance and seasonal dynamics of planktonic and benthic communities caused by hydrophysical and hydrochemical specifics: altitude above sea level, solar irradiance, water balance in the watershed basin, etc.

In our case, we found that, despite significant differences in the species composition of cyanobacteria from the two related lakes, the same cosmopolitan species represented toxigenic bacteria in them, as in most water bodies on the Earth. Previously, we reported on the *Microcystis*, *Dolichospermum* and *Snowella*-like cyanobacteria that contained MC synthetase genes [20,85], as well as on saxitoxin-producing *Nostoc* [119] in Lake Baikal. Since 2010, toxic *Dolichospermum* and *Microcystis* producing MCs in low concentration, presumably associated with the global temperature rise, were detected in the coastal zones and bays of Lake Baikal [20]. In other words, the toxigenic cyanobacteria in these lakes are cosmopolitan, which confirms the old age of the microcystin synthetase cluster.

The mass development of filamentous algae, death of endemic sponges, intensive growth and colonisation of toxic cyanobacteria on various substrates have been observed in Lake Baikal over the past decade. These features have become an “environmental crisis” caused by increased anthropogenic pressure, namely, the excessive supply of nutrients with rains and a deep influx of groundwater from the lake shores [120]. However, the present trophic state of the lake is oligotrophic based on hydrochemical parameters; signs of eutrophication were identified in the coastal zone of some areas of the lake [95]. Our observations and long-term monitoring of phytoplankton showed that the abundance of planktonic cyanobacteria in Lake Baikal was lower than 20 million cells L^−1^ not reaching the dangerous threshold [30]. At the same time, vegetation of the species *Dolichospermum* with a maximum abundance of up to 10 million cells L^−1^ has been regularly recorded in July and August since the 20th century [121]. Excessive growth of *Dolichospermum* cyanobacteria (*D*. *lemmermannii* as the dominant species) was first reported in the littoral zone on the western shore of the southern basin at the end of July and the beginning of August in 2019. The abundance of cyanobacteria in blooming spots varied within 7.2–72 million cells L^−1^, with 0.73–7.20 g m^−3^ of biomass attained [16].

Information about cyanobacterial blooms in large oligotrophic ecosystems (“oligotrophic blooms”) has appeared relatively recently; in some lakes, researchers observed mass development of mainly nontoxic diazotrophic cyanobacteria [14,15,122,123,124,125]. *Gloeotrichia echinulata* blooms were recorded in oligotrophic and mesotrophic lakes in the northeast of the United States [122], and prolific growths of *Dolichospermum*, *Aphanizomenon*, *Microcystis* and *Gloeotrichia* were observed in low-productive lakes in the province of Ontario [123]. The largest lakes of the Alps (Garda, Iseo, Como, and Maggiore) were progressively colonised by the species *D. lemmermannii* [124,125]. In the oligotrophic Lake Superior, often referred to as the most pristine of the Laurentian Great Lakes, the mass development of *D. lemmermannii* [14,15] was experienced. Recently, the North Patagonian lakes, notwithstanding their low nutrient concentration and productivity, also demonstrated cyanobacterial blooms, mainly constituted by toxigenic *Microcystis* and *Dolichospermum* species [126].

In Lake Khubsugul, we detected *D. lemmermannii* that had not been previously found there. It seems an intriguing finding presaging mass development of this species in the lake. The present survey did not reveal toxic genotypes of this species, apparently due to its low abundance (689 cells L^−1^). In this respect, further search for toxigenic *Dolichospermum* in the lake is needed. The occurrence of toxigenic *Dolichospermum* in Lake Khubsugul may be regarded as a trend for toxic algal blooms similar to Baikal events.

Under favourable conditions, toxigenic cyanobacteria pose a potential risk of producing cyanotoxins in concentration hazardous for humans and animals. Identifying the diversity of bloom-causing cyanobacterial species and revealing associated factors that stimulate these blooms are useful tools to predict and control them.

In Lake Khubsugul, anthropogenic eutrophication is one of the most controlled factors contributing to the mass development of cyanobacteria, with the appropriate regulation of economic, tourist and recreational activities, as well as animal husbandry, but it should not remain a major issue in the near future. The prevailing influence of global warming allows us to use the ecosystem of Lake Khubsugul as a model for studying the effect of climate change on the lake biota. It is assumed that the unique ecosystem of Lake Khubsugul would be affected by long-term warming and drying with a possible succession of hydrobionts species composition.

## 4. Conclusions

Based on our data on transparency, concentrations of mineral and organic compounds of phosphorus and nitrogen, and chlorophyll *a*, Lake Khubsugul may be attributed to oligotrophic lakes. The water in Lake Khubsugul is classified as clean in terms of faecal indicator microorganisms and the concentration of organic matter. The analysis of microbiomes showed small amounts of pathogenic and opportunistic bacteria of anthropogenic origin in the lake. In microbial communities, we identified the sequences of pathogenic and opportunistic bacteria of animal origin. The genetic and taxonomic diversity of cyanobacteria in the plankton and benthos of Lake Khubsugul is small, and the abundance of planktonic cyanobacteria is low. No unique and endemic species were encountered, mostly just cosmopolitan species. Diatoms and green algae dominated phytoplankton; picoplanktonic genera of the order Synechococcales prevailed among cyanobacteria, and members of the order Oscillatoriales predominated in the benthos. Synechococcales were subdominant, while Chroococcales and Nostocales were minor. We detected cyanobacteria containing MC synthetase genes, which belonged to the genera *Microcystis*, *Nostoc* and possibly *Snowella* in the plankton from the coastal zones of Lake Khubsugul. Cyanobacteria inhabiting the lake bottom produced five MC congeners, both common and rare. Toxins were not found in the pelagic plankton. MC concentration in benthic cyanobacteria from Lake Khubsugul is much lower than in other water bodies. At present, we suggest that anthropogenic loads on the lake ecosystem are minimal with no signs of cyanobacterial blooms. In the future, with slow changes in the global climate, considering the preservation of the current trophic state of the water body and relatively low anthropogenic activity, the risk of cyanobacterial blooms and toxin production is minimal, even in the presence of toxigenic genotypes and toxin-producing members. Apparently, there is always a potential source of occurrence and spread of toxic blooms in ancient oligotrophic lakes due to the existence of cyanobacteria containing toxin synthesis genes. In the future, under conditions favourable for mass vegetation of cyanobacteria, toxic blooms are possible. The presence of toxigenic and toxic cosmopolitan cyanobacteria in the ancient and oligotrophic Lake Khubsugul confirms the paleo origin of MC synthesis genes that appeared even before the formation of the lake itself.

## 5. Materials and Methods

### 5.1. Site Description

Lake Khubsugul is located in Central Asia on the territory of Mongolia. It lies between 50°30′ and 51°35′ N, 100°15′ and 100°40′ E. The basin of Lake Khubsugul is an extreme southwestern wing of the Baikal Rift Zone system, one of the largest rift zones on the Earth. Khubsugul is an ancient lake: the age of the sedimentary cover of the lake depression is 5.5 million years old [117]. The lake is situated at an altitude of 1645 m above sea level; its basin is trough-shaped with an area of 2760 km^2^, average width of 20.3 km, maximum width of 36.5 km, length of 136 km and water volume reaching 383.3 km^3^ [88,91]. The average depth of the lake is 139 m, and the maximum depth is 262 m. The pelagic zone occupies about 85% of the lake surface, and the littoral zone with a depth down to 50 m—15%.

The climate is continental, with extreme ranges of daily and annual air temperatures and a small amount of precipitation. The average annual air temperature is below zero (−4… −5 °C); the annual range of average monthly temperatures on the lake coast is 30–35 °C. In summer, the air temperature rises to 11–14 °C and changes during the day from 5 to 16 °C. The ice cover breaks in June, and the lake freezes in late November or early December; the ice thickness reaches 1.5 m [88]. The water in Lake Khubsugul is low-mineralised (210 to 230 mg L^−1^) and belongs to the bicarbonate class of calcium group, type 1. It has an alkaline reaction (pH 8.1 to 8.5), high oxygen concentration from the surface to the bottom in all seasons (8.10 to 11.43 mg L^−1^) and low CO_2_ in any season.

Forty-six rivers and streams flow into the lake, and only the Egiin Gol River flows out of the lake, which is a tributary of the Selenga River flowing into Lake Baikal. Therefore, there is a direct water connection between the two largest lakes in Central Asia. The amount of precipitation in the Khubsugul region is low: the maximum precipitation is in summer (100 to 300 mm), which is 60 to 70% of the annual amount.

### 5.2. Sampling

**Figure 9:**
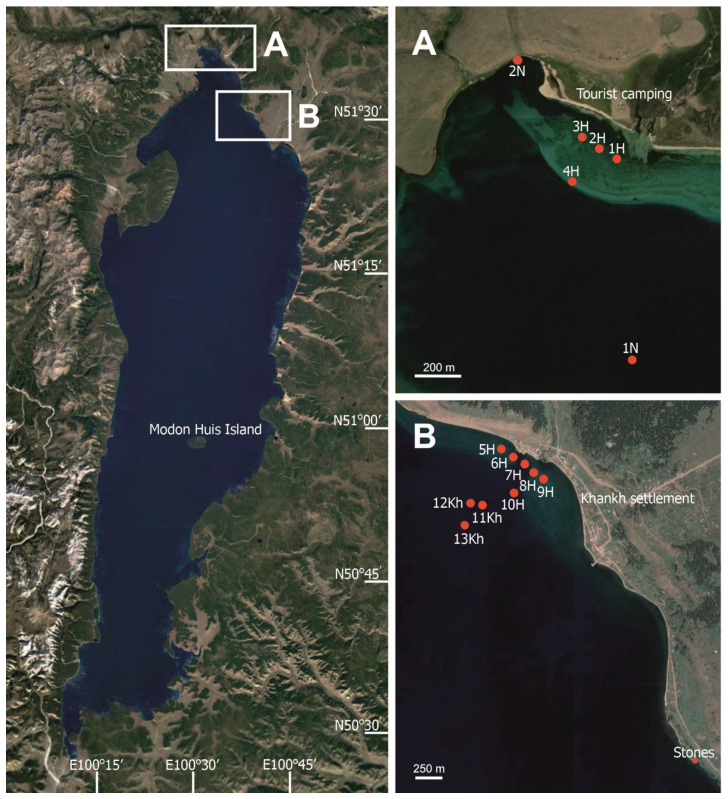
Map of Lake Khubsugul. Sites of sampling in 2017: The northern margin of the lake (**A**) and water area near Khankh settlement (**B**). Imagery © 2021 NASA, TerraMetrics, Map data © 2021 INEGI.

Water and biofilms from stones were sampled from 11 to 14 July 2017 in the northern part of Lake Khubsugul: on the eastern coast off the Khankh settlement and close to the northern margin of the lake (Figure 9). The water was sampled at four stations in the pelagic zone from the surface to 25 m depth (1N and 11–13Kh) and at ten stations in the littoral zone at 0 m (1H to 10H). The surface water was sampled with sterile bottles; water from the depths of 5, 10, 15 and 25 m was sampled with a Ruttner bathometer. Net sampling was also carried out using an Apstein net with a mesh size of 16 µm. The net was towed three to five times from depths of 1 to 5 m until a concentrated sample was obtained.

Biofilms formed on the stones were taken together with the stones from the lake bottom at a depth of 2 to 3 m (stations 2N and “Stones”) and transferred to the laboratory. The biofilms were then scraped from the surface of the stones with a sterile spatula and placed into sterile test tubes.

For the microscopic examination of phytoplankton, including cyanobacteria, samples were prepared by two methods. First, the samples were fixed with Lugol solution, concentrated by sedimentation and observed using light microscopy. Second, the samples were fixed with 4% formaldehyde (final concentration), filtered through polycarbonate filters with a pore size of 0.4 µm (Millipore, Burlington, MA, USA) and examined via epifluorescent microscopy. The net plankton and biofilms for light microscopy were also fixed with 4% formaldehyde. For ELISA, mass spectrometry and genetic analysis, net samples were frozen in liquid nitrogen. For genetic studies and determination of the chlorophyll concentration, unfixed samples of 1 L each from each layer were filtered in duplicate through polycarbonate filters with a pore size of 0.2 µm (Millipore, Burlington, MA, USA) and frozen. For microbiological analysis, the water samples were taken according to the ISO standard [127]. Simultaneously with sampling, water transparency was determined using a Secchi disk, and the temperature and pH were determined using a portable pH meter (Hanna Instruments, Woonsocket, RI, USA).

### 5.3. Sanitary-Microbiological Analysis

An assessment of the water quality in Lake Khubsugul was carried out according to the hygienic standards established by the sanitary rules and norms and methodological recommendations of the Russian Federation [68,69,70]. We determined the number of total coliforms, thermotolerant coliforms, enterococci, *Escherichia coli* and coliphages (Table S2).

The water quality was assessed based on the category of the water body. According to sanitary rules and regulations, water use category I includes water bodies or their areas used for drinking and domestic purposes, as well as for the water supply of food industry enterprises [69]. Water use category II includes water bodies or their areas used for recreational purposes, as well as localities within the settlement boundaries (Table S3).

### 5.4. Microscopy

Quantitative and qualitative analysis of phytoplankton and cyanobacteria was carried out using an Axio Imager M1 light microscope (Carl Zess, Jena, Germany), equipped with a BO 50 mercury lamp, three sets of filters (G 365, BP 450–490 and BP 546/12) and an AxioCam MRm camera. Algae and cyanobacteria were counted in duplicate in a 0.1 mL Nageotte chamber with magnifications of ×400 and ×1000. Biomass was calculated from algal abundance and individual cell volumes. To determine the biovolume, 30 cells of each species were measured. In case of using the filtration method and epifluorescent microscopy, cells were counted on 20 photomicrographs with the Image-Pro Plus 6.0 software (Media Cybernetics Inc., Silver Spring, MD, USA). Cyanobacteria were identified based on the autofluorescence of phycobilins, additional photosynthetic pigments, which were observed under a green optical filter (wavelength 540 nm). Identification was carried out according to the manuals [128,129,130].

### 5.5. Molecular Genetic Analysis

Total DNA was extracted from the samples using the DNA-sorb B kit, according to the manufacturer’s protocol (Central Research Institute of Epidemiology of Rospotrebnadzor, Moscow, Russia).

To search for and identify cyanobacteria containing microcystin synthesis genes, we used primers on the genes encoding microcystin synthetase (*mcy*). HepF and hepR primers were applied to detect the AMT domain of the *mcy*E gene [86]. The DNA of the toxin-producing strain, *Microcystis aeruginosa* CALU 972 (Botanical Institute collection, St. Petersburg State University, St. Petersburg, Russia), was used as a positive control. The amplicon length was 470 bp. The sequences were deposited in GenBank under accession numbers OM810332–OM810346.

Amplification and cloning were performed as described previously [19,20]. Determination of the nucleotide sequences of the cloned fragments (sequencing) according to the Sanger method was performed on a Beckman CEQ 8800 automatic sequencer (Beckman Coulter Inc., Brea, CA, USA). Unique sequences assigned as the closest relatives were included in the phylogenetic analysis. Amino acids were aligned in the Mega X software using the ClustalW algorithm [131]. A phylogenetic tree was constructed through Bayesian analysis using the MrBayes software (v. 3.2.6) [132]. Two independent Markov chain Monte Carlo (MCMC) analyses were launched for 25 million generations with 25% burn-in (rejection of initial generations) and four chains (one cold and three hot ones); the amino acid model was blosum62. The analysis was completed with an average standard deviation of the split frequencies: 0.02; the parameter potential scale reduction factor (PSRF+) was 1.000.

To study the taxonomic composition of microbial communities, the fragments of the 16S rRNA gene containing two variable regions V3–V4 were amplified using eubacterial primers 343F (CTCCTACGGRRSGCAG) and 806R (GGACTACNVGGGTWTCTAAT) [133]. Amplicon libraries were prepared for sequencing with the Nextera XT kit (Illumina, San Diego, CA, USA) and sequenced using MiSeq (Illumina, USA). Results were uploaded into NCBI SRA (BioProject #PRJNA820510). The quality of amplicon libraries was evaluated with FastQC software (http://www.bioinformatics.babraham.ac.uk/projects/fastqc; accessed on 1 January 2022); primers and spurious sequences were trimmed using cutadapt v1.14 [134]. The 16S rRNA gene fragments were aligned and taxonomically assigned using SILVA v.132 database with a confidence threshold of 80% [135] and clustered to OTUs at a 0.03 distance with mothur v.1.40.0 [136].

### 5.6. Enzyme-Linked Immunosorbent Assay

The concentration of toxins in samples was employed by ELISA using the Abraxis Microcystins-ADDA ELISA kit (Abraxis LLC, Warminster, PA, USA), according to the manufacturer’s instructions. Prior to ELISA testing biofilms collected from stony substrates (14 samples) and water with phytoplankton (20 samples) were lyophilised and then resuspended in water. Cyanobacterial material was lysed with QuickLyse (Abraxis LLC) and centrifuged to provide clarified extracts for ELISA. The results were processed with the RIDA^®^ SOFT Win software.

### 5.7. High Performance Liquid Chromatography-High Resolution Mass Spectrometry

Cell disintegration was carried out by a freeze-thaw cycle method, followed by grinding in a mortar after lyophilisation. MCs fraction was extracted from lyophilisate twice with 75% methanol for 60 min under sonication. The resulting extracts were combined, evaporated to dryness on a rotary evaporator at a temperature of 45 ± 2 °C and redissolved in methanol [18].

MCs were identified using the HPLC-HRMS/TOF system (Agilent 1200 HPLC chromatograph coupled with Agilent 6210 mass-spectrometer, Santa Clara, CA, USA). MCs were separated on a 150 × 2 mm Zorbax 300 SB-C18 column, using aqueous 0.1% formic acid (mobile phase A) and 0.1% formic acid in acetonitrile (mobile phase B). Gradient elution from 10% to 100% organic mobile phase for 40 min at 0.12 mL min^−1^ flow rate was applied. Detection was carried out in ESI+ mode, the *m/z* range from 100 to 3000 Da [137].

### 5.8. Hydrochemical Analysis

Concentration of the dissolved oxygen was measured using the method of Winkler as described previously [94]. Organic matter, total P and total N were determined in unfiltered water. Si, PO_4_^3−^, NO_3_^−^, NO_2_^−^ and NH_4_^+^ were analysed in water samples filtered through mixed cellulose ester membrane filters (Advantec, Tokyo, Japan), with a pore diameter 0.45 μm. Concentration of the nutrients was determined using a spectrophotometer UNICO-2100 (Dayton, NJ, USA) according to [138]: PO_4_^3−^ was identified by the Denigès–Atkins method in modification with tin chloride, NH_4_^+^ was detected by the indophenol method [139] and silica with a method based on measuring the intensity of the yellow colour accompanying the formation of the silicomolybdic acid complex. NO_3_^−^ and NO_2_^−^ concentration was measured by high performance liquid chromatography (EcoNova, Novosibirsk, Russia) with UV detection on an inverse-phase column modified with octadecyltrimethylammonium bromide [94,95]. Total N and P were measured spectrophotometrically after oxidation with persulfate. Permanganate oxidation index [140] and bichromate oxidation demand were used to assess organic substances content [141]. Chlorophyll *a* concentration was determined as described previously [66].

### 5.9. Statistical Analysis

The correlation matrix among biological and hydrochemical parameters was calculated using Spearman’s correlation coefficient. All *p*-values were adjusted for multiple testing with Benjamini-Hochberg (BH) false discovery rate (FDR) [142]; associations with a BH *p*-value < 0.05 were considered significant.

The Kruskal–Wallis test with *p*-value adjustment by the BH method was used to assess statistical difference between depth layers of water samples. Principal Component Analysis was performed on a log10 transformed data matrix of nutrients concentrations. Only factors with *p*-value < 0.05 were chosen for PCA.

All calculations were performed in the R software (v. 4.1.3) using corrplot [143] and plyr [144] packages.

## Figures and Tables

**Table 2:** Cyanobacteria of Lake Khubsugul based on light microscopy, HTS and the literature data. Planktonic species are highlighted in bold; grey colour mark species detected previously using microscopy [56,65].

No	Species by Microscopy	Number of OTUs ^1^	Closest Homologue in GenBank	Identity, %
1	***Anabaena*** **sp.**	-	-	-
2	*Calothrix parietina*	2	*C. parietina* 2T10	99.1, 97.7
3	*Chamaesiphon subglobosus*	1	*C. subglobosus* PCC 7430	99.1
4	*Chamaesiphon polonicus*	2	*C. polonicus* SAG 32.87	99.1, 99.7
5	*Chroococcus minutus*	12	*Pseudocapsa* sp. Ru3-14/*Aliterella gigantea* PJ102	97.2–99.1
6	*Chroococcus* sp.	12	*Pseudocapsa* sp. Ru3-14/*Aliterella gigantea* PJ102	97.2–99.1
7	***Coelosphaerium kuetzingianum***	-	-	-
8	***Dolichospermum lemmermannii***	-	-	-
9	*Gloeobacter* sp.	1	*Gloeobacter kilaueensis* JS1	99.3
10	*Leibleinia epiphytica*	15	*Timaviella circinata* GR4/*T*. *edaphica* Golos-9-1/*Timaviella* sp. Us-6-3	97.5–99.1
11	*Leptolyngbya* sp.	6	*Leptolyngbya* sp. CENA293	95.7–97.5
12	*Limnococcus limneticus*	-	-	-
13	*Nostoc* sp.	1	*Nostoc* sp. LEGE 04357	100
14	*Oscillatoria* sp.	-	-	-
15	***Planktothrix*** **sp.**	-	-	-
16	*Pseudanabaena galeata*	2	*Pseudanabaena frigida* ULC067	99.8, 97.8
17	***Pseudanabaena*** **sp.**	-	-	-
18	***Snowella rosea***	-	-	-
19	***S. lacustris***	-	-	-
20	*Tolypothrix mongolica*	-	-	-
21	***Trichodesmium lacustre***	-	-	-
22	***Tychonema tenue***	1	*Tychonema* sp. SAG 23.89/*M. pseudautumnalis* Ak1609/*P. autumnale* CCALA 143	100
23	*Rivularia coadunata*	1	*Rivularia* sp. VP4-08	99.1
24	-	2	*Altericista variichlora* CALU 1173	98.7, 98.9
25	-	1	*Shackletoneilla antarctica* ANT.L18.1	99.6
26	-	1	*Nodosilinea* sp. 19D10hp	99.8
27	-	1	*Jaaginema geminatum* SAG 1459-8	100
28	-	1	*Pseudanabaena foetida* TNS-AL-57779	100
29	-	6	*Stenomitos frigidus* ANT.LMA.1, ACT684	98–100
30	-	3	*Phormidesmis priestleyi* ANT.LPR2.6	99.1–100
31	-	1	*Scytonematopsis contorta* HA4292-MV4	99.8

^1^ OTUs—operational taxonomic units.

## Data Availability

PRJNA613961 freshwater mixed marker sequence reads are available online. URL: https://dataview.ncbi.nlm.nih.gov/object/PRJNA613961?reviewer=6nqhjaovurg89onscgc6sn6br6 Last access date: 14 April 2021. Release date: 23 April 2024.

## Supplementary Materials

The following supporting information can be downloaded at: https://www.mdpi.com/article/10.3390/toxins15030213/s1, Figure S1: number of pair-end reads, OTUs and alpha-diversity indices for bacterial communities in the samples from Lake Khubsugul; Figure S2: unrooted phylogenetic tree obtained with MrBayes, based on the alignment of *mcy*E gene sequences of cyanobacteria. Sequences from Lake Khubsugul are in bold. Bayesian posterior probabilities are indicated near their nodes. The scale bar shows 0.02 estimated substitutions per site; Figure S3: total MC concentration detected by ELISA in biofilms from stony substrates sampled in the coastal zone near Khankh settlement (st. “Stones”). Error bars represent standard deviation. Figure S4: extracted ion current chromatogram of microcystins from the Lake Khubsugul, integrated sample “Stones”; Figure S5: boxplots for nutrient concentrations in different depth water layers in Lake Khubsugul. Non-parametric Kruskal–Wallis test was executed to assess if there is any significant difference between the average nutrient concentrations in different depth layers of water. Abbreviations: A—abundance, B—biomass; Table S1: cyanobacteria species composition, abundance and biomass in biofilms on stones (n = 8) from Lake Khubsugul; Table S2: faecal indicator microorganisms in the water from Lake Khubsugul sampled on 11 to 14 July 2017; Table S3: guideline values of water safety indicators in surface water according to normative documents of the Russian Federation [68,69,70]; Table S4: results for the Kruskal–Wallis test based on depth factor dependence.

## References

[1] Knoll A.H., Barghoorn E.S. (1977). Archean microfossils showing cell division from the Swaziland system of South Africa. Science.

[2] Schopf J.W. (1993). Microfossils of the early Archean Apex Chert: New evidence of the antiquity of life. Science.

[3] Paerl H.W., Otten T.G., Kudela R. (2018). Mitigating the expansion of harmful algal blooms across the freshwater-to-marine continuum. Environ. Sci. Technol..

[4] Huisman J., Codd G.A., Paerl H.W., Ibelings B.W., Verspagen J.M.H., Visser P.M. (2018). Cyanobacterial blooms. Nat. Rev. Microbiol..

[5] Paerl H.W. (2018). Mitigating toxic planktonic cyanobacterial blooms in aquatic ecosystems facing increasing anthropogenic and climatic pressures. Toxins.

[6] Wood S.A., Kelly L.T., Bouma-Gregson K., Humbert J.F., Laughinghouse H.D., Lazorchak J., McAllister T.G., McQueen A., Pokrzywinski K., Puddick J. (2020). Toxic benthic freshwater cyanobacterial proliferations: Challenges and solutions for enhancing knowledge and improving monitoring and mitigation. Freshw. Biol..

[7] Wells M.L., Karlson B., Wulff A., Kudela R., Trick C., Asnaghi V., Berdalet E., Cochlan W., Davidson K., De Rijcke M. (2020). Future HAB science: Directions and challenges in a changing climate. Harmful Algae.

[8] Tanvir R.U., Hu Z., Zhang Y., Lu J. (2021). Cyanobacterial community succession and associated cyanotoxin production in hypereutrophic and eutrophic freshwaters. Environ. Pollut..

[9] Bhayani K., Paliwal C., Ghosh T., Mishra S. (2018). Nutra-cosmeceutical potential of pigments from microalgae. Sunscreens: Source, Formulations, Efficacy and Recommendations.

[10] Paliwal C., Nesamma A.A., Jutur P.P. (2019). Sustainable downstream processing of microalgae for in-dustrial application. Industrial Scope with High-Value Bio-Molecules from Microalgae.

[11] Paerl H.W., Otten T.G. (2013). Harmful cyanobacterial blooms: Causes, consequences, and controls. Microb. Ecol..

[12] Paerl H.W., Xu H., McCarthy M.J., Zhu G., Qin B., Li Y., Gardner W.S. (2011). Controlling harmful cyanobacterial blooms in a hyper-eutrophic lake (Lake Taihu, China): The need for a dual nutrient (N & P) management strategy. Water Res..

[13] Chorus I., Falconer I.R., Salas H.J., Bartram J. (2000). Health risks caused by freshwater cyanobacteria in recreational waters. J. Toxicol. Environ. Health Part B Crit. Rev..

[14] Sterner R.W., Reinl K.L., Lafrancois B.M., Brovold S., Miller T.R. (2020). A first assessment of cyanobacterial blooms in oligotrophic Lake Superior. Limnol. Oceanogr..

[15] Reinl K.L., Sterner R.W., Austin J.A. (2020). Seasonality and physical drivers of deep chlorophyll layers in Lake Superior, with implications for a rapidly warming lake. J. Great Lakes Res..

[16] Bondarenko N.A., Tomberg I.V., Shirokaya A.A., Belykh O.I., Tikhonova I.V., Fedorova G.A., Netsvetaeva O.G., Eletskaya E.V., Timoshkin O.A. (2021). *Dolichospermum lemmermannii* (Nostocales) bloom in world’s deepest Lake Baikal (East Siberia): Abundance, toxicity and factors influencing growth. Limnol. Freshw. Biol..

[17] Belykh O.I., Tikhonova I.V., Kuzmin A.V., Sorokovikova E.G., Fedorova G.A., Khanaev I.V., Sherbakova T.A., Timoshkin O.A. (2016). First detection of benthic cyanobacteria in Lake Baikal producing paralytic shellfish toxins. Toxicon.

[18] Belykh O.I., Fedorova G.A., Kuzmin A.V., Tikhonova I.V., Timoshkin O.A., Sorokovikova E.G. (2017). Microcystins in cyanobacterial biofilms from the littoral zone of Lake Baikal. Moscow Univ. Biol. Sci. Bull..

[19] Belykh O.I., Gladkikh A.S., Tikhonova I.V., Kuz’min A.V., Mogil’nikova T.A., Fedorova G.A., Sorokovnikova E.G. (2015). Identification of cyanobacterial producers of shellfish paralytic toxins in Lake Baikal. Mikrobiologiia.

[20] Belykh O.I., Gladkikh A.S., Sorokovikova E.G., Tikhonova I.V., Butina T.V. (2015). Identification of toxic cyanobacteria in Lake Baikal. Dokl. Biochem. Biophys..

[21] Pearson L., Mihali T., Moffitt M., Kellmann R., Neilan B. (2010). On the chemistry, toxicology and genetics of the cyanobacterial toxins, microcystin, nodularin, saxitoxin and cylindrospermopsin. Mar. Drugs.

[22] Plaas H.E., Paerl H.W. (2021). Toxic cyanobacteria: A growing threat to water and air quality. Environ. Sci. Technol..

[23] Omidi A., Esterhuizen-Londt M., Pflugmacher S. (2018). Still challenging: The ecological function of the cyanobacterial toxin microcystin–What we know so far. Toxin Rev..

[24] Preece E.P., Hardy F.J., Moore B.C., Bryan M. (2017). A review of microcystin detections in Estuarine and Marine waters: Environmental implications and human health risk. Harmful Algae.

[25] Bouaïcha N., Miles C.O., Beach D.G., Labidi Z., Djabri A., Benayache N.Y., Nguyen-Quang T. (2019). Structural diversity, characterization and toxicology of microcystins. Toxins.

[26] Janssen E.M.L. (2019). Cyanobacterial peptides beyond microcystins—A review on co-occurrence, toxicity, and challenges for risk assessment. Water Res..

[27] Chorus I., Welker M. (2021). Toxic Cyanobacteria in Water.

[28] Chen L., Giesy J.P., Adamovsky O., Svirčev Z., Meriluoto J., Codd G.A., Mijovic B., Shi T., Tuo X., Li S.-C. (2021). Challenges of using blooms of *Microcystis* spp. in animal feeds: A comprehensive review of nutritional, toxicological and microbial health evaluation. Sci. Total Environ..

[29] WHO (2020). Cyanobacterial Toxins: Microcystins. Guidelines for Drinking-Water Quality and Guidelines for Safe Recreational Water Environments. WHO/HEP/ECH/WSH/2020.6.

[30] WHO (2003). Guidelines for Safe Recreational Water Environments. Volume 1: Coastal and Fresh Water.

[31] WHO (2017). Guidelines for Drinking-Water Quality: Fourth Edition Incorporating the First Addendum.

[32] Dittmann E., Neilan B.A., Erhard M., Von Döhren H., Börner T. (1997). Insertional mutagenesis of a peptide synthetase gene that is responsible for hepatotoxin production in the cyanobacterium *Microcystis aeruginosa* PCC 7806. Mol. Microbiol..

[33] Nishizawa T., Asayama M., Fujii K., Harada K., Shirai M. (1999). Genetic analysis of the peptide synthetase genes for a cyclic heptapeptide microcystin in *Microcystis* spp.. J. Biochem..

[34] Nishizawa T., Ueda A., Asayama M., Fujii K., Harada K.I., Ochi K., Shirai M. (2000). Polyketide synthase gene coupled to the peptide synthetase module involved in the biosynthesis of the cyclic heptapeptide microcystin. J. Biochem..

[35] Tillett D., Dittmann E., Erhard M., Von Döhren H., Börner T., Neilan B.A. (2000). Structural organization of microcystin biosynthesis in Microcystis aeruginosa PCC7806: An integrated peptide-polyketide synthetase system. Chem. Biol..

[36] Christiansen G., Fastner J., Erhard M., Börner T., Dittmann E. (2003). Microcystin biosynthesis in *Planktothrix*: Genes, evolution, and manipulation. J. Bacteriol..

[37] Rouhiainen L., Vakkilainen T., Siemer B.L., Buikema W., Haselkorn R., Sivonen K. (2004). Genes coding for hepatotoxic heptapeptides (microcystins) in the cyanobacterium *Anabaena* strain 90. Appl. Environ. Microbiol..

[38] Fewer D.P., Wahlsten M., Österholm J., Jokela J., Rouhiainen L., Kaasalainen U., Rikkinen J., Sivonen K. (2013). The genetic basis for O-acetylation of the microcystin toxin in cyanobacteria. Chem. Biol..

[39] Rounge T.B., Rohrlack T., Nederbragt A.J., Kristensen T., Jakobsen K.S. (2009). A genome-wide analysis of nonribosomal peptide synthetase gene clusters and their peptides in a *Planktothrix rubescens* strain. BMC Genom..

[40] Heck K., Alvarenga D.O., Shishido T.K., Varani A.M., Dörr F.A., Pinto E., Rouhiainen L., Jokela J., Sivonen K., Fiore M.F. (2018). Biosynthesis of microcystin hepatotoxins in the cyanobacterial genus *Fischerella*. Toxicon.

[41] Shishido T.K., Jokela J., Humisto A., Suurnäkki S., Wahlsten M., Alvarenga D.O., Sivonen K., Fewer D.P. (2019). The biosynthesis of rare homo-amino acid containing variants of microcystin by a benthic cyanobacterium. Mar. Drugs.

[42] Kaplan A., Harel M., Kaplan-Levy R.N., Hadas O., Sukenik A., Dittmann E. (2012). The languages spoken in the water body (or the biological role of cyanobacterial toxins). Front. Microbiol..

[43] Holland A., Kinnear S. (2013). Interpreting the possible ecological role(s) of cyanotoxins: Compounds for competitive advantage and/or physiological aide?. Mar. Drugs.

[44] Rantala A., Fewer D.P., Hisbergues M., Rouhiainen L., Vaitomaa J., Börner T., Sivonen K. (2004). Phylogenetic evidence for the early evolution of microcystin synthesis. Proc. Natl. Acad. Sci. USA.

[45] Burford M.A., Carey C.C., Hamilton D.P., Huisman J., Paerl H.W., Wood S.A., Wulff A. (2020). Perspective: Advancing the research agenda for improving understanding of cyanobacteria in a future of global change. Harmful Algae.

[46] Paerl H.W., Barnard M.A. (2020). Mitigating the global expansion of harmful cyanobacterial blooms: Moving targets in a human- and climatically-altered world. Harmful Algae.

[47] Barruffa A.S., Sposito V., Faggian R. (2021). Climate change and cyanobacteria harmful algae blooms: Adaptation practices for developing countries. Mar. Freshw. Res..

[48] Bartlett S.L., Brunner S.L., Klump J.V., Houghton E.M., Miller T.R. (2018). Spatial analysis of toxic or otherwise bioactive cyanobacterial peptides in Green Bay, Lake Michigan. J. Great Lakes Res..

[49] Millie D.F., Fahnenstiel G.L., Dyble J., Pigg R., Rediske R., Klarer D.M., Litaker R.W., Tester P.A. (2008). Influence of environmental conditions on late-summer cyanobacterial abundance in Saginaw Bay, Lake Huron. Aquat. Ecosyst. Health Manag..

[50] Perri K.A., Sullivan J.M., Boyer G.L. (2015). Harmful algal blooms in Sodus Bay, Lake Ontario: A comparison of nutrients, marina presence, and cyanobacterial toxins. J. Great Lakes Res..

[51] Watson S.B., Miller C., Arhonditsis G., Boyer G.L., Carmichael W., Charlton M.N., Confesor R., Depew D.C., Höök T.O., Ludsin S.A. (2016). The re-eutrophication of Lake Erie: Harmful algal blooms and hypoxia. Harmful Algae.

[52] Chaffin J.D., Mishra S., Kane D.D., Bade D.L., Stanislawczyk K., Slodysko K.N., Jones K.W., Parker E.M., Fox E.L. (2019). Cyanobacterial blooms in the central basin of Lake Erie: Potentials for cyanotoxins and environmental drivers. J. Great Lakes Res..

[53] Zhang D., Liao Q., Zhang L., Wang D., Luo L., Chen Y., Zhong J., Liu J. (2015). Occurrence and spatial distributions of microcystins in Poyang Lake, the largest freshwater lake in China. Ecotoxicology.

[54] Feng H., Clara T., Huang F., Wei J., Yang F. (2019). Identification and characterization of the dominant *Microcystis* sp. cyanobacteria detected in Lake Dong Ting, China. J. Toxicol. Environ. Health.

[55] Olokotum M., Mitroi V., Troussellier M., Semyalo R., Bernard C., Montuelle B., Okello W., Quiblier C., Humbert J.F. (2020). A review of the socioecological causes and consequences of cyanobacterial blooms in Lake Victoria. Harmful Algae.

[56] Kozhova O.M., Kobanova G.I., Goulden C.E., Sitnikova T., Gelhaus J., Boldgiv B. (2006). Phytoplankton of Lake Hövsgöl. The Geology, Biodiversity and Ecology of Lake Hövsgöl.

[57] Sodnom N., Losev N. (1976). Natural Conditions and Resources of Hovsgol Region.

[58] Zagorenko G.F., Losev N.F., Tsevgmid D. (1972). New Data about several algae of Lake Khubsugul. Natural Conditions and Resources of Hovsgol Region.

[59] Zagorenko G.F., Kozhova O.M., Batmunh J., Losev N.F. (1973). Structure and ecological data of summer phytoplankton of Lake Hovsgol in 1971. Natural Conditions and Resources of Hovsgol Region.

[60] Kozhova O.M., Zagorenko G.F., Ladejtschikova V.K. (1977). Peculiarities of annual and season dynamics of phytoplankton in Lake Khubsugul. Hydrobiol. J..

[61] Kozhov M.M., Antipova H.L., Vasilyeva G.L., Nikolaeva E.P. (1965). On the plankton of Lake Khubsugul (Kosogol). Tr. Limnol. Inst..

[62] Kozhova O.M., Izmest’eva L.R., Erbaeva E.A. (1994). A review of the hydrobiology of Lake Khubsugul (Mongolia). Hydrobiologia.

[63] Kozhova O.M., Kobanova G.I., Izmestyeva L.R. (2000). Summer Phytoplankton of Lake Khubsugul (Mongolia). Hydrobiol. J..

[64] Hindák F., Zagorenko G.F. (1992). Contribution to the knowledge of the species composition of summer phytoplankton of Lake Hubsugul, Mongolia. Folia Geobot. Phytotaxon..

[65] Zagorenko G.F., Prozorov B.A. (1983). A new species of the genus *Tolypothrix* (Scytonemataceae, Cyanophyta) from the Khubsugul Lake in Mongolia. Bot. Zhurnal.

[66] Belykh O.I., Sorokovikova E.G., Tikhonova I.V., Fedotov A.F. (2005). Abundance, morphological diversity, and spatial distribution of autotrophic picoplankton in Lake Hovsgol (Mongolia). Aquat. Ecosyst. Health Manag..

[67] Likens G.E., Leith H., Whittaker R. (1975). Primary production of inland aquatic ecosystems. Primary Production of the Biosphere.

[68] (1999). On the Sanitary and Epidemiological Welfare of the Population (with the Amendments and Additions).

[69] (2021). Hygienic Standards and Requirements for Ensuring the Safety and (or) Harmlessness of Environmental Factors for Humans.

[70] (2004). Control Methods. Biological and Microbiological Factors. Sanitary-Microbiological and Sanitary-Parasitological Analysis of Surface Water Bodies.

[71] Cruz A.T., Cazacu A.C., Allen C.H. (2007). *Pantoea agglomerans*, a plant pathogen causing human disease. J. Clin. Microbiol..

[72] Müller H.E., Brenner D.J., Fanning G.R., Grimont P.A.D., Kampfer P. (1996). Emended description of *Buttiauxella agrestis* with recognition of six new species of *Buttiauxella* and two new species of *Kluyvera*: *Buttiauxella ferragutiae* sp. nov., *Buttiauxella gaviniae* sp. nov., *Buttiauxella brennerae* sp. nov., *Buttiauxella izardii* sp. nov., *Buttiauxella noackiae* sp. nov., *Buttiauxella warmboldiae* sp. nov., *Kluyvera cochleae* sp. nov., and *Kluyvera georgiana* sp. nov. Int. J. Syst. Bacteriol..

[73] Ramamurthy T., Chowdhury G., Pazhani G.P., Shinoda S. (2014). *Vibrio fluvialis*: An emerging human pathogen. Front. Microbiol..

[74] Saraoui T., Leroi F., Björkroth J., Pilet M.F. (2016). *Lactococcus piscium*: A psychrotrophic lactic acid bacterium with bioprotective or spoilage activity in food—A review. J. Appl. Microbiol..

[75] Clements K.D., Sutton D.C., Choat J.H. (1989). Occurrence and characteristics of unusual protistan symbionts from surgeonfishes (Acanthuridae) of the Great Barrier Reef, Australia. Mar. Biol..

[76] Wang X.Q., Zhao D.L., Shen L.L., Jing C.L., Zhang C.S., Meena V.S. (2018). Application and mechanisms of *Bacillus subtilis* in biological control of plant disease. Role of Rhizospheric Microbes in Soil: Stress Management and Agricultural Sustainability.

[77] Alebouyeh M., Gooran O.P., Azimi-Rad M., Tajbakhsh M., Tajeddin E., Jahani S.S., Nazemalhosseini M.E., Zali M.R. (2011). Fatal sepsis by bacillus circulans in an immunocompromised patient. Iran. J. Microbiol..

[78] Becker K., Heilmann C., Peters G. (2014). Coagulase-negative staphylococci. Clin. Microbiol. Rev..

[79] Waters J.L., Ley R.E. (2019). The human gut bacteria *Christensenellaceae* are widespread, heritable, and associated with health. BMC Biol..

[80] Egan M., Dempsey E., Ryan C.A., Ross R.P., Stanton C. (2021). The Sporobiota of the Human Gut. Gut Microbes.

[81] Cai S., Dong X. (2010). *Cellulosilyticum ruminicola* gen. nov., sp. nov., isolated from the rumen of yak, and reclassification of *Clostridium lentocellum* as *Cellulosilyticum lentocellum* comb. nov. Int. J. Syst. Evol. Microbiol..

[82] Gerritsen J., Umanets A., Staneva I., Hornung B., Ritari J., Paulin L., Rijkers G.T., de Vos W.M., Smidt H. (2018). *Romboutsia hominis* sp. nov., the first human gut-derived representative of the genus romboutsia, isolated from ileostoma effluent. Int. J. Syst. Evol. Microbiol..

[83] Jumas-Bilak E., Carlier J.P., Jean-Pierre H., Mory F., Teyssier C., Gay B., Campos J., Marchandin H. (2007). *Acidaminococcus intestini* sp. nov., isolated from human clinical samples. Int. J. Syst. Evol. Microbiol..

[84] D’Auria G., Galán J.C., Rodríguez-Alcayna M., Moya A., Baquero F., Latorre A. (2011). Complete genome sequence of *Acidaminococcus intestini* RYC-MR95, a gram-negative bacterium from the phylum Firmicutes. J. Bacteriol..

[85] Ivacheva M.A., Tikhonova I.V., Sorokovikova E.G., Krasnopeev A.Y., Potapov S.A., Choydash B., Belykh O.I. (2016). Microcystin-producing cyanobacteria in the benthos of Lake Baikal. Bull. Irkutsk. State Univ. Ser. Biol..

[86] Jungblut A.D., Neilan B.A. (2006). Molecular identification and evolution of the cyclic peptide hepatotoxins, microcystin and nodularin, synthetase genes in three orders of cyanobacteria. Arch. Microbiol..

[87] Romanenko V.D., Oksijuk O.P., Zhukinsky V.N., Stolberg F.V., Lavrik V.I. (1990). Ecological Impact Assessment of Hydrotechnical Constructions on Water Bodies.

[88] Bogoyavlensky B. (1989). Atlas of Lake Hubsugul.

[89] Tarasova E.N., Mamontova E.A., Mamontov A.A., Goreglyad A.V., Tsypukova S.S., Tkatchenko L.L. (2017). The spatial and time change ability of chemical composition of water of Lake Hovsgol (Mongolia). Environ. Chem..

[90] Tarasova E.N. (1998). Components of trophic status in water of Lake Baikal, the Lake Hovsgol and the Lake Teletskoe. Contemp. Probl. Ecol..

[91] Goulden C.E., Sitnikova T.Y., Gelhaus J., Boldgiv B. (2006). The Geology, Biodiversity and Ecology of Lake Hövsgöl (Mongolia).

[92] Sorokovikova L.M., Tomberg I.V., Sinyukovich V.N., Molozhnikova E.V., Khodzher T.V. (2019). Low water level in the Selenga River and reduction of silica input to Lake Baikal. Inland Waters.

[93] (2020). Integrated Livelihoods Improvement and Sustainable Tourism in Khuvsgul Lake National Park Project. MON 9873. Water Quality Monitoring Program for Khuvsgul Lake National Park–Final Consultant Report.

[94] Khodzher T.V., Domysheva V.M., Sorokovikova L.M., Golobokova L.P., Mueller L., Sheudshen A.K., Eulenstein F. (2016). Methods for monitoring the chemical composition of Lake Baikal water. Novel Methods for Monitoring and Managing Land and Water Resources in Siberia.

[95] Khodzher T.V., Domysheva V.M., Sorokovikova L.M., Sakirko M.V., Tomberg I.V. (2017). Current chemical composition of Lake Baikal water. Inland Waters.

[96] Oyungerel B. (2011). Reflection of the global warming in the change of the Lake Khuvsgul. Bull. Buryat State Univ. Biol. Geogr..

[97] Bezuijen M.R., Russell M., Zomer R.J., Enkhtaivan D. (2020). Building the Climate Change Resilience of Mongolia’s Blue Pearl: A Case Study of Khuvsgul Lake National Park.

[98] Zhang P., Jeong J.H., Yoon J.H., Kim H., Simon Wang S.Y., Linderholm H.W., Fang K., Wu X., Chen D. (2020). Abrupt shift to hotter and drier climate over inner East Asia beyond the tipping point. Science.

[99] Nandintsetseg B., Greene J.S., Goulden C.E. (2007). Trends in extreme daily precipitation and temperature near Lake Hövsgöl, Mongolia. Int. J. Climatol..

[100] Aboal M., Puig M.Á. (2005). Intracellular and dissolved microcystin in reservoirs of the river Segura basin, Murcia, SE Spain. Toxicon.

[101] Mohamed Z.A., El-Sharouny H.M., Ali W.S.M. (2006). Microcystin production in benthic mats of cyanobacteria in the Nile River and irrigation canals, Egypt. Toxicon.

[102] Sorokovikova E.G., Belykh O.I., Gladkikh A.S., Kotsar O.V., Tikhonova I.V., Timoshkin O.A., Parfenova V.V. (2013). Diversity of cyanobacterial species and phylotypes in biofilms from the littoral zone of Lake Baikal. J. Microbiol..

[103] Dorofeyuk N.I., Tsetsegma D., Ulzijkhutag N., Gunin P.D. (2002). Algae Flora in Mongolia.

[104] Barboza G.F.O., Gorlach-Lira K., Sassi C.F.C., Sassi R. (2017). Microcystins production and antibacterial activity of cyanobacterial strains of *Synechocystis*, *Synechococcus* and *Romeria* from water and coral reef organisms (Brazil). Rev. Biol. Trop..

[105] Gagunashvili A.N., Andrésson Ó.S. (2018). Distinctive characters of *Nostoc* genomes in cyanolichens. BMC Genom..

[106] Sivonen K., Namikoshi M., Evans W.R., Färdig M., Carmichael W.W., Rinehart K.L. (1992). Three new microcystins, cyclic heptapeptide hepatotoxins, from *Nostoc* sp. strain 152. Chem. Res. Toxicol..

[107] Sivonen K., Carmichael W.W., Namikoshi M., Rinehart K.L., Dahlem A.M., Niemela S.I. (1990). Isolation and characterization of hepatotoxic microcystin homologs from the filamentous freshwater cyanobacterium *Nostoc* sp. strain 152. Appl. Environ. Microbiol..

[108] Dodds W.K., Gudder D.A., Mollenhauer D. (1995). The ecology of *Nostoc*. J. Phycol..

[109] Jones A.C., Monroe E.A., Eisman E.B., Gerwick L., Sherman D.H., Gerwick W.H. (2010). The unique mechanistic transformations involved in the biosynthesis of modular natural products from marine cyanobacteria. Nat. Prod. Rep..

[110] Oksanen I., Jokela J., Fewer D.P., Wahlsten M., Rikkinen J., Sivonen K. (2004). Discovery of rare and highly toxic microcystins from lichen-associated cyanobacterium *Nostoc* sp. strain IO-102-I. Appl. Environ. Microbiol..

[111] Kleinteich J., Puddick J., Wood S.A., Hildebrand F., Laughinghouse H.D., Pearce D.A., Dietrich D.R., Wilmotte A. (2018). Toxic cyanobacteria in Svalbard: Chemical diversity of microcystins detected using a liquid chromatography mass spectrometry precursor ion screening method. Toxins.

[112] Wood S.A., Mountfort D., Selwood A.I., Holland P.T., Puddick J., Cary S.C. (2008). Widespread Distribution and Identification of Eight Novel Microcystins in Antarctic Cyanobacterial Mats. Appl. Environ. Microbiol..

[113] Kleinteich J., Wood S.A., Puddick J., Schleheck D., Kupper F.C., Dietrich D. (2013). Potent toxins in Arctic environments--presence of saxitoxins and an unusual microcystin variant in Arctic freshwater ecosystems. Chem. Biol. Interact..

[114] Quiblier C., Wood S., Echenique-Subiabre I., Heath M., Villeneuve A., Humbert J.-F. (2013). A review of current knowledge on toxic benthic freshwater cyanobacteria—ecology, toxin production and risk management. Water Res..

[115] Mats V.D. (2012). The sedimentary fill of the Baikal Basin: Implications for rifting age and geodynamics. Russ. Geol. Geophys..

[116] Kuz’min M.I., Yarmolyuk V.V. (2006). Mountain growth and climatic variations in the Earth’s history. Russ. Geol. Geophys..

[117] Fedotov A.P., De Batist M., Pouls T. (2006). Tectonic evolution of the southwestern wall of the Baikal Rift Zone. Dokl. Earth Sci..

[118] Logatchev N.A., Antoshenko-Olenev I.V., Bazarov D.B., Galkin V.I., Goldurev G.S., Endrikhinskij A.S., Zolatorev A.G., Sizikov A.I., Ufimzev G.F. (1974). The Uplands of the West Baikal and Trans-Baikal Regions.

[119] Zubkov I.N., Kuzmin A.V., Tikhonova I.V., Belykh O.I., Smirnov V.I., Ivanov A.V., Shagun V.A., Grachev M.A., Fedorova G.A. (2018). Method for Determination of Saxitoxins Using Hplc-Ms with 2,4-Dinitrophenylhydrazine Precolumn Derivatization. Proc. Univ. Appl. Chem. Biotechnol..

[120] Timoshkin O.A., Moore M.V., Kulikova N.N., Tomberg I.V., Malnik V.V., Shimaraev M.N., Troitskaya E.S., Shirokaya A.A., Sinyukovich V.N., Zaitseva E.P. (2018). Groundwater contamination by sewage causes benthic algal outbreaks in the littoral zone of Lake Baikal (East Siberia). J. Great Lakes Res..

[121] Popovskaya G.I. (2000). Ecological monitoring of phytoplankton in Lake Baikal. Aquat. Ecosyst. Health Manag..

[122] Carey C.C., Weathers K.C., Cottingham K.L. (2008). *Gloeotrichia echinulata* blooms in an oligotrophic lake: Helpful insights from eutrophic lakes. J. Plankton Res..

[123] Winter J.G., Desellas A.M., Fletcher R., Heintsch L., Morley A., Nakamoto L., Utsumi K. (2011). Algal blooms in Ontario, Canada: Increases in reports since 1994. Lake Reserv. Manag..

[124] Callieri C., Bertoni R., Contesini M., Bertoni F. (2014). Lake level fluctuations boost toxic cyanobacterial “oligotrophic blooms”. PLoS ONE.

[125] Salmaso N., Capelli C., Shams S., Cerasino L. (2015). Expansion of bloom-forming *Dolichospermum lemmermannii* (Nostocales, Cyanobacteria) to the deep lakes south of the Alps: Colonization patterns, driving forces and implications for water use. Harmful Algae.

[126] Nimptsch J., Woelfl S., Osorio S., Valenzuela J., Moreira C., Ramos V., Castelo-Branco R., Leão P.N., Vasconcelos V. (2016). First record of toxins associated with cyanobacterial blooms in oligotrophic North Patagonian lakes of Chile-a genomic approach. Int. Rev. Hydrobiol..

[127] (2006). Water Quality—Sampling for Microbiological Analysis.

[128] Komárek J., Anagnostidis K., Ettl H., Gerloff J., Heynig H., Mollenhauer D. (1999). Süβwasserflora von Mitteleuropa, Band 19/1. Cyanoprocaryota. Teil/Part 1: Chroococcales.

[129] Komárek J., Anagnostidis K., Büdel B., Krienitz L., Gärtner G., Schagerl M. (2005). Süβwasserflora von Mitteleuropa. Bd. 19/2. Cyanoprokaryota. Teil/Part 2: Oscillatoriales.

[130] Komárek J., Büdel B., Gärtner G., Krienitz L., Schagerl M. (2013). Süßwasserflora von Mitteleuropa, Bd. 19/3. Cyanoprokaryota. Teil/Part 3: Heterocytous genera.

[131] Kumar S., Stecher G., Li M., Knyaz C., Tamura K. (2018). MEGA X: Molecular Evolutionary Genetics Analysis across computing platforms. Mol. Biol. Evol..

[132] Huelsenbeck J.P., Ronquist F. (2001). MRBAYES: Bayesian inference of phylogenetic trees. Bioinformatics.

[133] Caporaso J.G., Lauber C.L., Walters W.A., Berg-Lyons D., Huntley J., Fierer N., Owens S.M., Betley J., Fraser L., Bauer M. (2012). Ultra-high-throughput microbial community analysis on the Illumina HiSeq and MiSeq platforms. ISME J..

[134] Martin M. (2011). Cutadapt removes adapter sequences from high-throughput sequencing reads. EMBnet J..

[135] Quast C., Pruesse E., Yilmaz P., Gerken J., Schweer T., Yarza P., Peplies J., Glöckner F.O. (2013). The SILVA ribosomal RNA gene database project: Improved data processing and web-based tools. Nucleic Acids Res..

[136] Schloss P.D., Westcott S.L., Ryabin T., Hall J.R., Hartmann M., Hollister E.B., Lesniewski R.A., Oakley B.B., Parks D.H., Robinson C.J. (2009). Introducing mothur: Open-source, platform-independent, community-supported software for describing and comparing microbial communities. Appl. Environ. Microbiol..

[137] Belykh O.I., Sorokovikova E.G., Fedorova G.A., Kaluzhnaya O.V., Korneva E.S., Sakirko M.V., Sherbakova T.A. (2011). Presence and genetic diversity of microcystin-producing cyanobacteria (*Anabaena* and *Microcystis*) in Lake Kotokel (Russia, Lake Baikal Region). Hydrobiologia.

[138] Wetzel R.G., Likens G.E. (2000). Limnological Analyses.

[139] Bolleter W.T., Bushman C.J., Tidwell P.W. (1961). Spectrophotometric Determination of Ammonia as Indophenol. Anal. Chem..

[140] (1993). Water Quality—Determination of Permanganate Index.

[141] Baird R.B., Eaton A.D., Rice E.W. (2017). Standard Methods for the Examination of Water and Wastewater.

[142] Benjamini Y., Hochberg Y. (1995). Controlling the False Discovery Rate: A Practical and Powerful Approach to Multiple Testing. J. R. Stat. Soc. Ser. B Stat. Methodol..

[143] Wei T., Simko V. R Package ‘Corrplot’: Visualization of a Correlation Matrix. (Version 0.92). https://github.com/taiyun/corrplot.

[144] Wickham H. (2011). The split-apply-combine strategy for data analysis. J. Stat. Softw..

