# Supplementary material for: Water Quality, Toxicity and Diversity of Planktonic and Benthic Cyanobacteria in Pristine Ancient Lake Khubsugul (Hövsgöl), Mongolia
Source: Toxins (Basel). 2023 Mar 10;15(3):213. doi: 10.3390/toxins15030213 (PMC10053237; doi:10.3390/toxins15030213)
Supplement: Supplementary file 1 [file toxins-15-00213-s001.zip › toxins-2250594-supplementary.pdf]

Supplementary Materials for Article

Water Quality, Toxicity and Diversity of Planktonic and Benthic Cyanobacteria in Pristine Ancient Lake Khubsugul (Hövsgöl), Mongolia

Olga I. Belykh [1*], Ekaterina G. Sorokovikova [1*], Irina V. Tomberg [1], Galina A. Fedorova [1], Anton V. Kuzmin [1], Andrey Yu. Krasnopeev [1], Mariya Yu. Suslova [1], Sergey A. Potapov [1], Tatiana I. Belykh [2], Jadambaa Norovsuren [3], Agnia D. Galachyants [1], Irina V. Tikhonova [1]

Table S1. Cyanobacteria species composition, abundance and biomass in biofilms on stones (n=8) from Lake Khubsugul.

Species	Abundance, Ind. m^{-2}		Biomass, g m^{-2}	
	**Mean**	**SD**	**Mean**	**SD**
Rivularia coadunata	58115942	5039303	4407	303
Leptolyngbya sp.	17758152	3481410	3	0,49
Nostoc sp.	5448370	2431507	157	70
Chamaesiphon subglobosus	851449	224186	0,02	0,01
Total	81580616	6415337	4566	319

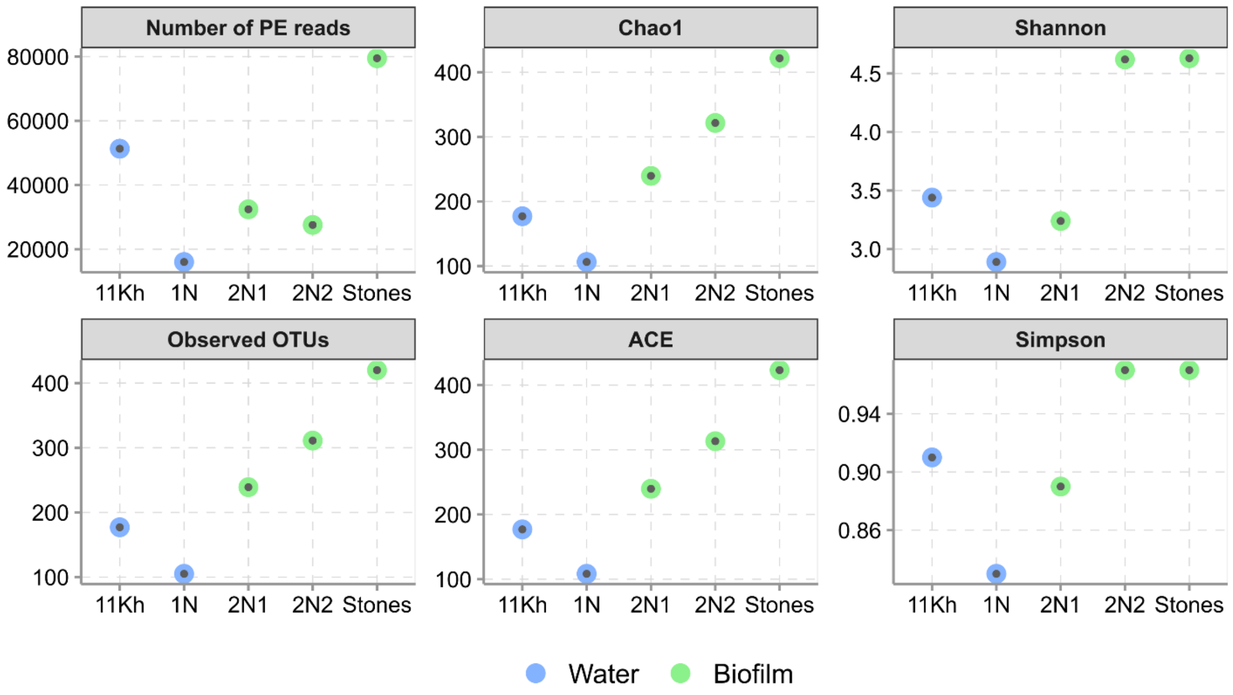

Figure S1. Number of pair-end reads, OTUs, and alpha-diversity indices for bacterial communities in the samples from Lake Khubsugul.

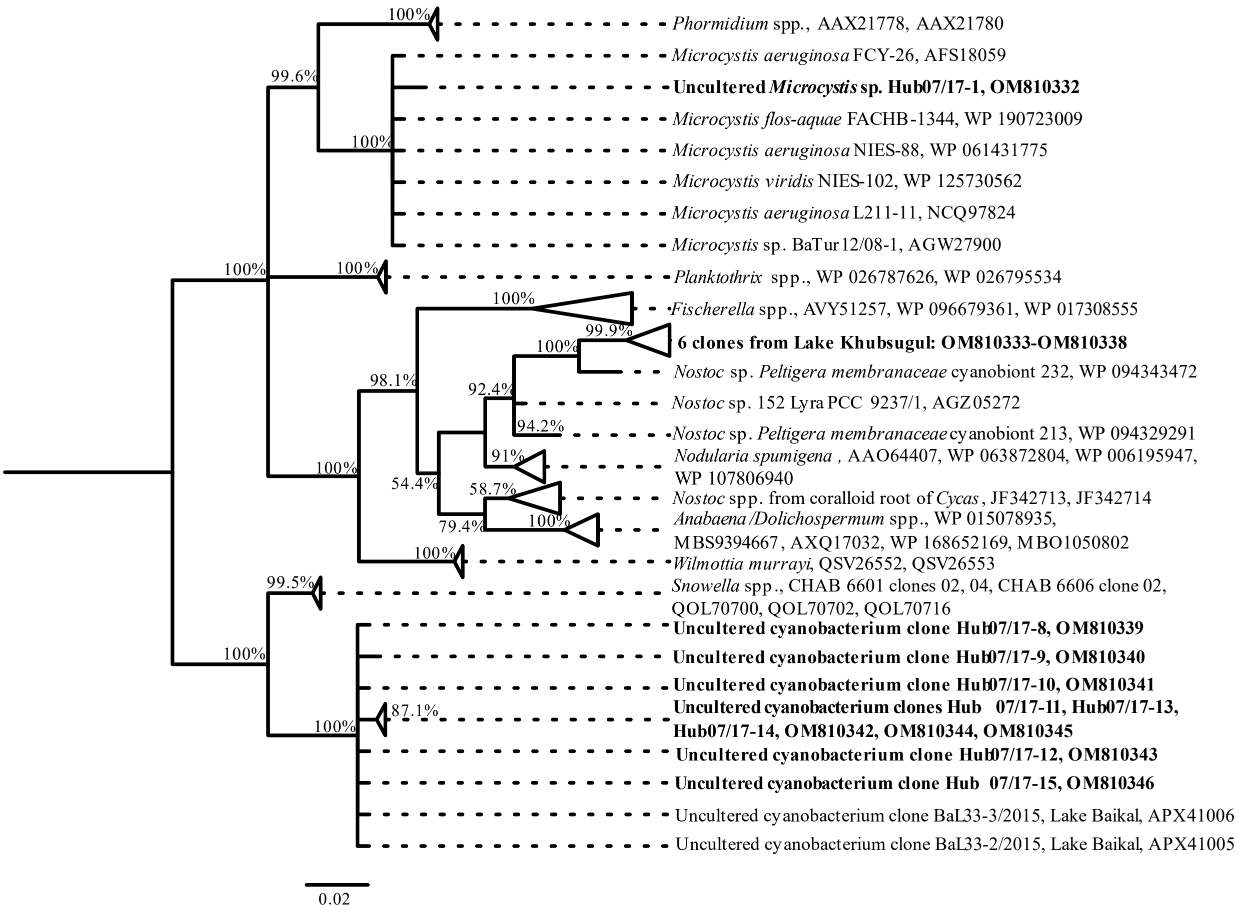

Figure S2. Unrooted phylogenetic tree obtained with MrBayes based on the alignment of *mcy*E gene sequences of cyanobacteria. Sequences from Lake Khubsugul are in bold. Bayesian posterior probabilities are indicated near their nodes. The scale bar shows 0.02 estimated substitutions per site.

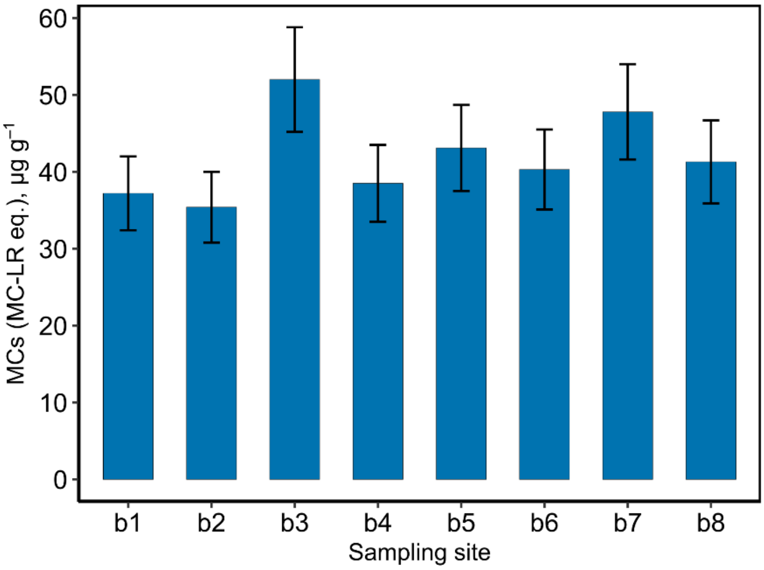

Figure S3. Total MC concentration detected by ELISA in biofilms from stony substrates sampled in the coastal zone near Khankh settlement (st. "Stones"). Error bars represent standard deviation.

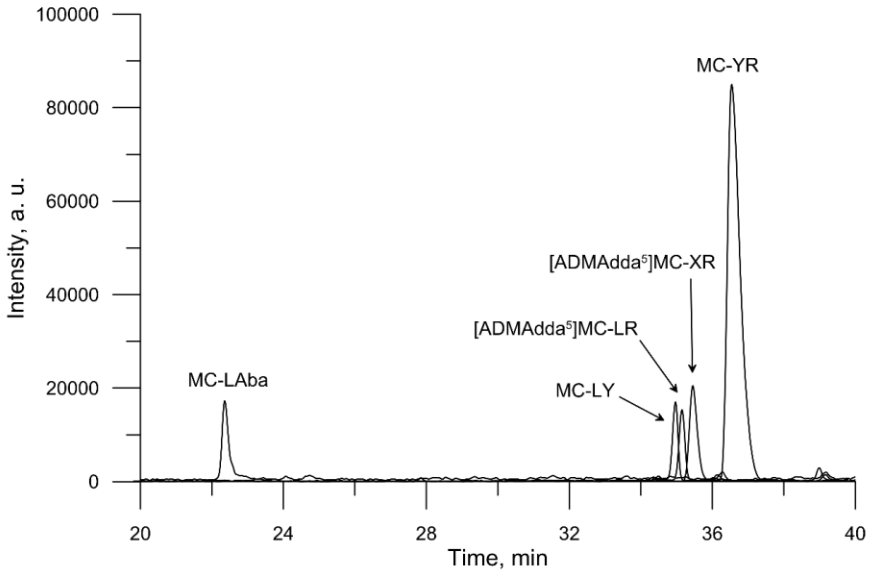

Figure S4. Extracted ion current chromatogram of microcystins from the Lake Khubsugul, integrated sample "Stones".

Table S2. Faecal indicator microorganisms in the water from Lake Khubsugul sampled on 11 to 14 July 2017.

No	Sample	Total coliforms, CFU in 100 ml	Thermotolerant coliforms, CFU in 100 ml	*E. coli*, CFU in 100 ml	Enterococci, CFU in 100 ml	Coliphages, PFU in 100 ml
1	1H, 0 m	0	0	0	4 ± 1	0
2	2H, 0 m	5 ± 1	4 ± 0	4 ± 0	6 ± 0	0
3	3H, 0 m	1 ± 0	1 ± 0	1 ± 0	0	0
4	4H, 0 m	0	0	0	1 ± 0	0
5	5H, 0 m	54 ± 2	0	0	2 ± 0	0
6	6H, 0 m	2 ± 1	2 ± 1	2 ± 1	1 ± 0	0
7	7H, 0 m	0	0	0	0	0
8	8H, 0 m	2 ± 1	2 ± 1	2 ± 1	0	0
9	9H, 0 m	24 ± 2	0	0	0	0
10	10H, 0 m	0	0	0	0	0
11	1N, 0 m	0	0	0	0	0
12	1N, 5 m	0	0	0	0	0
13	1N, 10 m	0	0	0	0	0
14	1N, 15 m	0	0	0	0	0
15	1N, 25 m	0	0	0	0	0
16	11Kh, 0 m	0	0	0	2 ± 1	0
17	11Kh, 5 m	0	0	0	0	0
18	11Kh, 10 m	0	0	0	2 ± 1	0
19	11Kh, 15 m	2 ± 1	2 ± 1	2 ± 1	0	0
20	11Kh, 25 m	2 ± 1	2 ± 1	2 ± 1	0	0

Note: CFU – colony-forming units; PFU – plaque-forming units.

Table S3. Guideline values of water safety indicators in surface water according to normative documents of the Russian Federation [68–70].

Water use category	Total coliforms, CFU in 100 ml	Thermotolerant coliforms, CFU in 100 ml	*E. coli*, CFU in 100 ml	Enterococci, CFU in 100 ml	Coliphages, PFU in 100 ml
Category I	≤ 1000	≤ 100	≤ 100	≤ 100	≤ 10
Category II	≤ 500	≤ 100	≤ 100	≤ 10	≤ 10

Note: CFU – colony-forming units; PFU – plaque-forming units.

Table S4. Results for Kruskal-Wallis test based on depth factor dependence.

Factor	n	statistic	df	p.adj	p.adj.signif
Ec_25°C	20	16.729	4	0.00218	**
Si	20	10.271	4	0.0361	*
N_{org}	20	17.414	4	0.00161	**
TN	20	16.484	4	0.00243	**
DIP	20	17.386	4	0.00163	**
P_{org}	20	16.171	4	0.0028	**
TP	20	16.514	4	0.0024	**
COD_{Cr}	20	18.286	4	0.00109	**
COD_{Mn}	20	10.529	4	0.0324	*
Chl_a	20	11.143	4	0.025	*
Cyclotella_ocellata_A	20	17.513	4	0.00154	**
Mychonastes_minusculus_A	20	13.900	4	0.00762	**
Dinobryon_sociale_A	20	13.657	4	0.00847	**
Monoraphidium_contortum_A	20	11.037	4	0.0262	*
Others_A	20	14.314	4	0.00636	**
Total_A	20	17.171	4	0.00179	**
Cyclotella_ocellata_B	20	17.513	4	0.00154	**
Mychonastes_minusculus_B	20	13.910	4	0.00759	**
Dinobryon_sociale_B	20	13.657	4	0.00847	**
Monoraphidium_contortum_B	20	10.829	4	0.0286	*
Others_B	20	15.953	4	0.00308	**
Total_B	20	13.657	4	0.00847	**

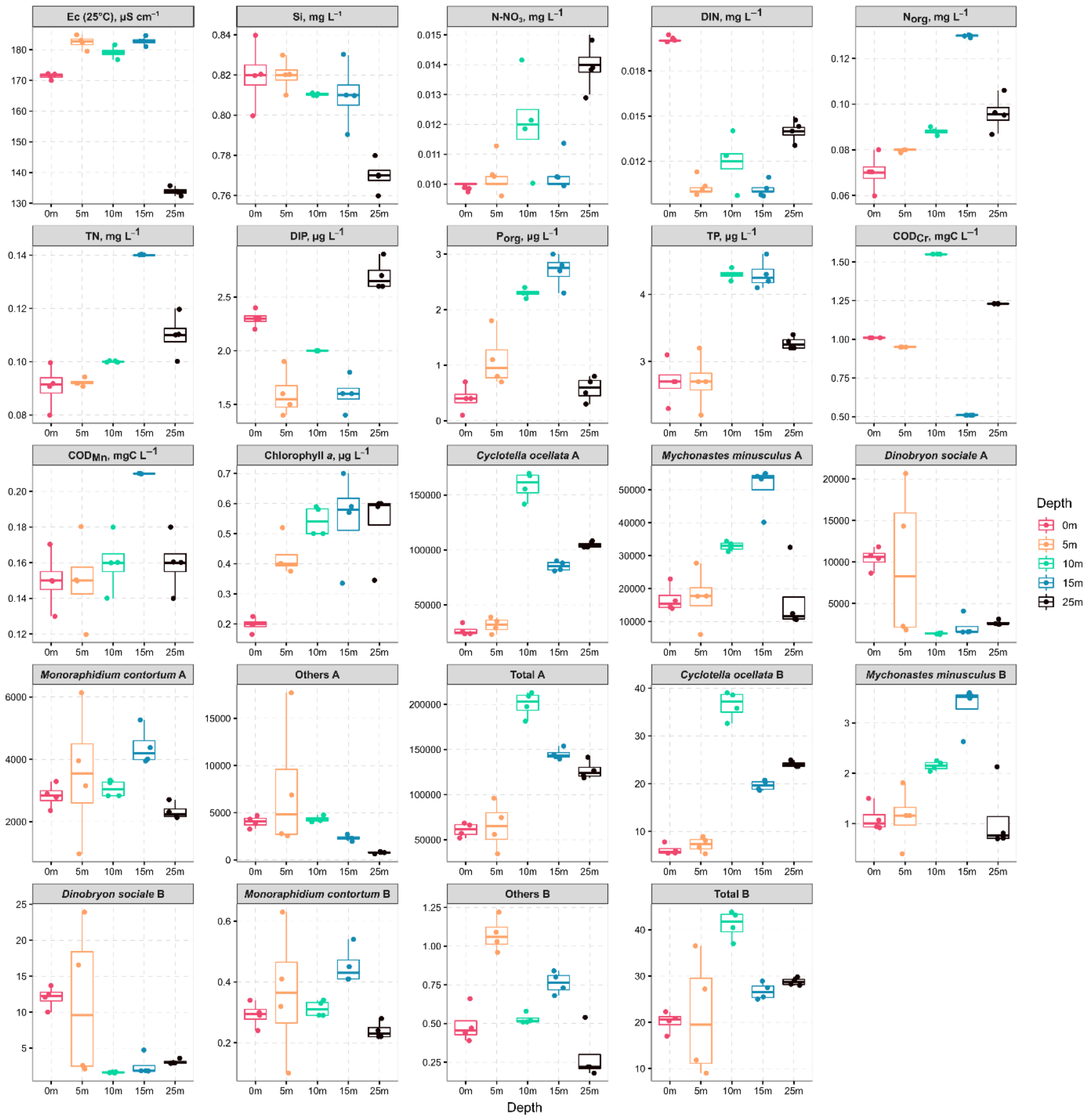

Figure S5. Boxplots for nutrient concentrations in different depth water layers in Lake Khubsugul. Non-parametric Kruskal-Wallis test was executed to assess if there is any significant difference between the average nutrient concentrations in different depth layers of water. Abbreviations: A – abundance, B – biomass.
